# Improving the Conductivity of Amide-Based Small Molecules through Enhanced Molecular Packing and Their Application as Hole Transport Mediators in Perovskite Solar Cells

**DOI:** 10.1021/acsaem.3c01988

**Published:** 2023-11-08

**Authors:** Eman A. A. Alkhudhayr, Dumitru Sirbu, Miriam Fsadni, Benjamin Vella, Bening T. Muhammad, Paul G. Waddell, Michael R. Probert, Thomas J. Penfold, Toby Hallam, Elizabeth A. Gibson, Pablo Docampo

**Affiliations:** † Energy Materials Laboratory, Newcastle University, Newcastle upon Tyne NE1 7RU, U.K.; ⊥ Chemistry, Newcastle University, Newcastle upon Tyne NE1 7RU, U.K.; ⊗ Physics, School of Mathematics, Statistics and Physics, Newcastle University, Newcastle upon Tyne NE1 7RU, U.K.; ‡ School of Chemistry, University of Glasgow, Glasgow G12 8QQ, U.K.; § Department of Physics, College of Science, King Faisal University, Al Ahsa 31982, Saudi Arabia; ○ School of Natural and Environmental Sciences, Bedson Building,Newcastle University, Newcastle upon Tyne NE1 7RU, U.K.

**Keywords:** hole-transporting materials, Perovskite solar cells, low cost, amide, molecular packing

## Abstract

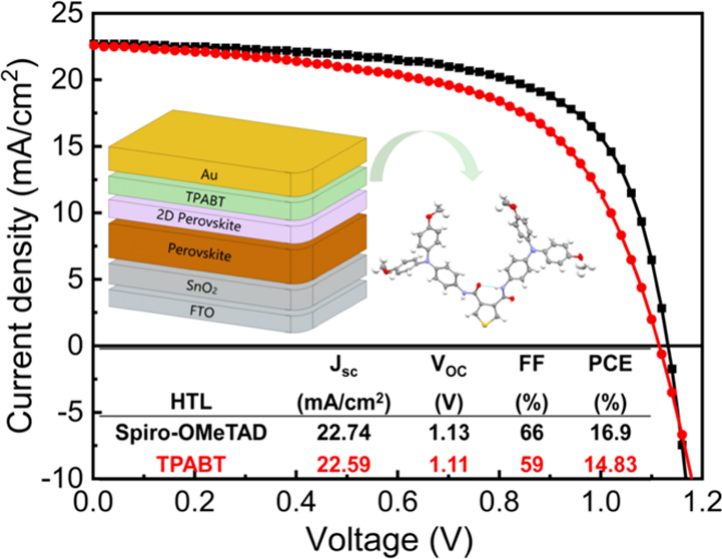

Organic–inorganic hybrid halide perovskite solar cells (PSCs) have attracted substantial attention from the photovoltaic research community, with the power conversion efficiency (PCE) already exceeding 26%. Current state-of-the-art devices rely on Spiro-OMeTAD as the hole-transporting material (HTM); however, Spiro-OMeTAD is costly due to its complicated synthesis and expensive product purification, while its low conductivity ultimately limits the achievable device efficiency. In this work, we build upon our recently introduced family of low-cost amide-based small molecules and introduce a molecule (termed TPABT) that results in high conductivity values (∼10^–5^ S cm^–1^ upon addition of standard ionic additives), outperforming our previous amide-based material (EDOT-Amide-TPA, ∼10^–6^ S cm^–1^) while only costing an estimated $5/g. We ascribe the increased optoelectronic properties to favorable molecular packing, as shown by single-crystal X-ray diffraction, which results in close spacing between the triphenylamine blocks. This, in turn, results in a short hole-hopping distance between molecules and therefore good mobility and conductivity. In addition, TPABT exhibits a higher bandgap and is as a result more transparent in the visible range of the solar spectrum, leading to lower parasitic absorption losses than Spiro-OMeTAD, and has increased moisture stability. We applied the molecule in perovskite solar cells and obtained good efficiency values in the ∼15% range. Our approach shows that engineering better molecular packing may be the key to developing high-efficiency, low-cost HTMs for perovskite solar cells.

## Introduction

Perovskite solar cells (PSCs) are classified under the third generation of photovoltaics^1^ and are considered a promising emerging technology due to their excellent properties: high charge carrier mobility, intense light absorption ability, defect tolerance, and compatibility with low-cost, solution-based fabrication processes.^2^ In the past decade, constant efforts have been made to improve the device engineering for perovskite solar cells, leading to a remarkable increase in the power conversion efficiency (PCE) from 3.81% in 2009^3^ to over 26%.^4^ PSCs typically contain two electrodes, a layer of hole-transporting material (HTM), a layer of electron-transporting material (ETM), and a perovskite-based light-absorbing layer.^5,6^ When the PSC devices are illuminated, the photogenerated electrons and holes in the perovskite are transferred to the ETM and HTM and then collected by the front and back electrodes.^7^

The HTM has played a key part in the development of PSCs, and its performance can determine the efficiency and stability. In “regular” structured PSCs, the HTM is placed on top of the perovskite layer and it performs a variety of roles, which include: (i) proficient extraction of the photogenerated holes, including facilitating electron transport to the counter electrode and hindering electron transfer back to the anode; (ii) promoting device stability by inhibiting direct contact between the perovskite and electrode (typically gold, silver, and aluminum) and safeguarding against the dispersal of the electrode into the perovskite layer; and (iii) ensuring uniform coverage of the perovskite layer to minimize the charge recombination losses at the perovskite and electrode interface.^5^ To maximize the PSC performance, the properties of the HTM must satisfy certain fundamental requirements, which include a small offset in energy between the highest occupied molecular orbital (HOMO) of the HTM and the perovskite valence band (VB), an adequate hole mobility, and both thermal and photochemical stability.

Many HTMs have been analyzed and integrated into PSCs, including organic and inorganic materials. The most common organic examples are 2,2′,7,7′-tetrakis[*N*,*N*-di(4-methoxyphenyl)amino]-9,9′-spirobifluorene (Spiro-OMeTAD), polytriarylamine (PTAA), poly(3,4-ethylenedioxythiophene) polystyrenesulfonate (PEDOT–PSS), and poly(3-hexylthiophene-2,5-diyl) P3HT.^8^ Multistep synthetic methods, involving metal-catalyzed cross-coupling reactions and stringent reaction conditions, are required to synthesize these materials, followed by lengthy purification.^9^ This makes HTMs like spiro-OMeTAD complicated and expensive to manufacture on a large scale, with anticipated costs in the range of ∼90 $/g.^10^ This represents a significant contribution to the total cost of the device and, consequently, may hinder the commercial success of perovskite solar cells.

Spiro-OMeTAD, like many organic semiconductors employed as HTMs, has low conductivity and mobility in its pristine form, and thus requires ionic dopants to increase its hole mobility.^11,12^ These additives commonly include mixtures of lithium bis(trifluoromethanesulfonyl)imide (LiTFSI) and tris(2-(1*H*-pyrazol-1-yl)-4-*tert*-butylpyridine)cobalt(III) tri[bis(trifluoromethane)sulfonimide] (FK209). Doping results from a partial oxidation of the HTM, introducing a small number of single-occupancy molecular orbitals within the HTL matrix. The presence of a number of deepened energy levels within the matrix lowers the energetic barrier toward charge hopping, greatly increasing the mobility.^12,13^ High mobility in the HTL is paramount to deliver high PCEs as efficient charge extraction is necessary to reduce undesirable electron–hole recombination at the interface with the perovskite layer.

To this end, there has also been much research into dopant-free hole transport materials, which have appreciably high intrinsic mobility. Here, triarylamine-based molecules, i.e., TAD derivatives, have shown remarkable hole mobility even without the influence of dopants,^14,15^ resulting in values above those found for doped spiro-OMeTAD.^16^ This is an important pathway toward surpassing the limitations of the current crop of record-breaking devices, as the low hole mobility of spiro-OMeTAD is a clear limiting factor in state-of-the-art perovskite solar cells.^17,18^

We have recently introduced condensation chemistry as a low-cost approach, i.e., in the range of 1–10 $/g, to high-quality organic molecules with a lower environmental impact.^13,19−21^ This includes aromatic amides, which have additional advantages over conventional C–C coupling because of the dipole nature of the bond arising from the difference in the electronegativity of the nitrogen and oxygen atom. Consequently, stronger interactions within and between the molecules could be promoted through the ability of amide groups to form intramolecular and intermolecular hydrogen bonds.^22^ Amide groups are also planar in geometry and have a high rotational barrier due to their partial double bond character.^23^ These properties can lead to denser molecular packing in the HTM layer. There may also be opportunities to take advantage of the possibility to form monodentate complexes between the oxygen atoms of the amide group with metal atoms, for example, building lead or lithium adducts when applied in PSCs.^24^ These chemical and structural properties of amide molecules make these compounds highly promising for applications in solar devices.

In the present work, we describe a new HTM (TPABT), based on familiar building blocks linked together by functional amides, and its application in PSCs. For a nonconjugated material, this HTM demonstrates the exceptional properties of high conductivity and charge carrier mobility and a blue-shifted onset of absorption that avoids competition for light with the perovskite layer. These observations show that conjugation through the main chain is not important for materials with good charge transfer properties. Amide bonds in TPABT have the potential to coordinate with Li^+^ ions, which leads to an increase in the conductivity up to ca. 10^–5^ S cm^–1^ upon addition of standard ionic additives, outperforming our previous amide-based molecule, EDOT-Amide-TPA. We used TPABT to fabricate PSCs and obtained devices with PCE ∼ 15%. Our HTM TPABT is superior to Spiro-OMeTAD in terms of optical transparency and low cost, making this a key milestone in developing future low-cost HTMs.

## Results and Discussion

### Synthesis

**Figure 1:**
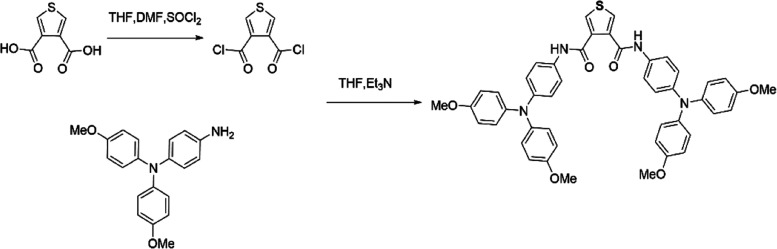
Reaction scheme and molecular structure of TPABT.

TPABT was synthesized by condensing thiophene β-dicarbonyl chloride and 4-amino-4′,4′′-dimethoxytriphenylamine, as illustrated in Figure 1. The structure of the product was confirmed by ^1^H and ^13^C NMR spectroscopies (Figures S1 and S2, Supporting Information). While the singlet at 10.33 ppm confirms the formation of an amide bond, the integral of 12 indicates the methoxy group (3.80 ppm) is in line with two triphenylamine units attached to the thiophene moiety. The successful synthesis was further corroborated by high-resolution mass spectrometry analysis showing the [M + H]^+^ cation (Figure S3, Supporting Information).

**Figure 2:**
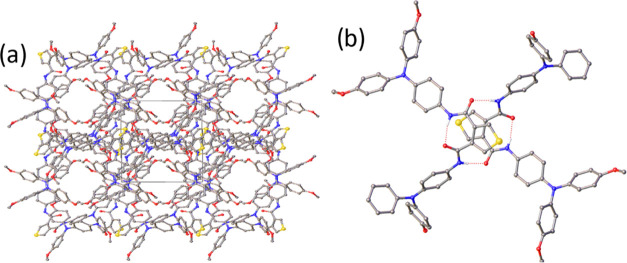
(a) View of the structure of TPABT-CHCl_3_ in the [001] direction highlighting the solvent-accessible channels. Hydrogen atoms and solvent molecules were omitted for clarity. (b) Typical hydrogen-bonded dimer of TPABT molecules as observed in each TPABT structure.

TPABT crystals of suitable quality for single-crystal X-ray diffraction measurements were grown by slow evaporation of the solvent (chloroform, chlorobenzene) or slow diffusion of an antisolvent into the TPABT solution (diethyl ether and tetrahydrofuran; hexane and chloroform). In all cases, the TPABT crystallized as a solvate; two polymorphs of the chloroform monosolvate (TPABT-CHCl_3_, and TPABT2-CHCl_3_), one chlorobenzene solvate (TPABT-CB), and one in which there is a 3:1 ratio of diethyl ether and tetrahydrofuran (TPABT-THF). It should be noted that crystallization from the CB/CHCl_3_ mixture yields the chlorobenzene solvate of TPABT identical to the structure obtained by evaporation of the neat chlorobenzene solution. In the case of TPABT-CHCl_3_, continuous solvent-accessible channels are formed along the crystallographic [001] direction (Figure 2). In the structure of TPABT-THF, the solvent molecules occupy the space between layers of TPABT molecules in the crystallographic (001) plane, while the solvent molecules in both TPABT2-CHCl_3_ and TPABT-CB occupy discrete pockets formed in the space between the TPA groups of the main residue. Chlorobenzene and chloroform are used as the solvent mixture during the spin-coating process when TPABT is deposited as the HTM layer in perovskite solar devices. Chlorobenzene has been used as an antisolvent for preparing perovskite films in perovskite solar cells. There is a limited choice of solvents that are appropriate to dissolve the TPABT. The perovskite must not be soluble in the solvent since the HTM layer is on top of the perovskite layer in the standard configuration in perovskite solar devices.

In each structure, the conformation of the TPABT molecule is constrained by an intramolecular hydrogen bond formed between the two amide groups. This appears to have the effect of reducing the distance between the two TPA units, as the average N2···N4 distance is ca. 12.1 Å compared to 18 Å calculated for the EDOT-amide-TPA. The shortest N2···N4 distance is observed in TPABT2-CHCl_3_ (ca. 10.9 Å). In addition, all four crystal structures form a hydrogen-bonded dimer of TPABT molecules, effectively reducing the distance between the TPA groups of neighboring molecules to ca. 12.1 Å (Figure 2).

### Computations

**Figure 3:**
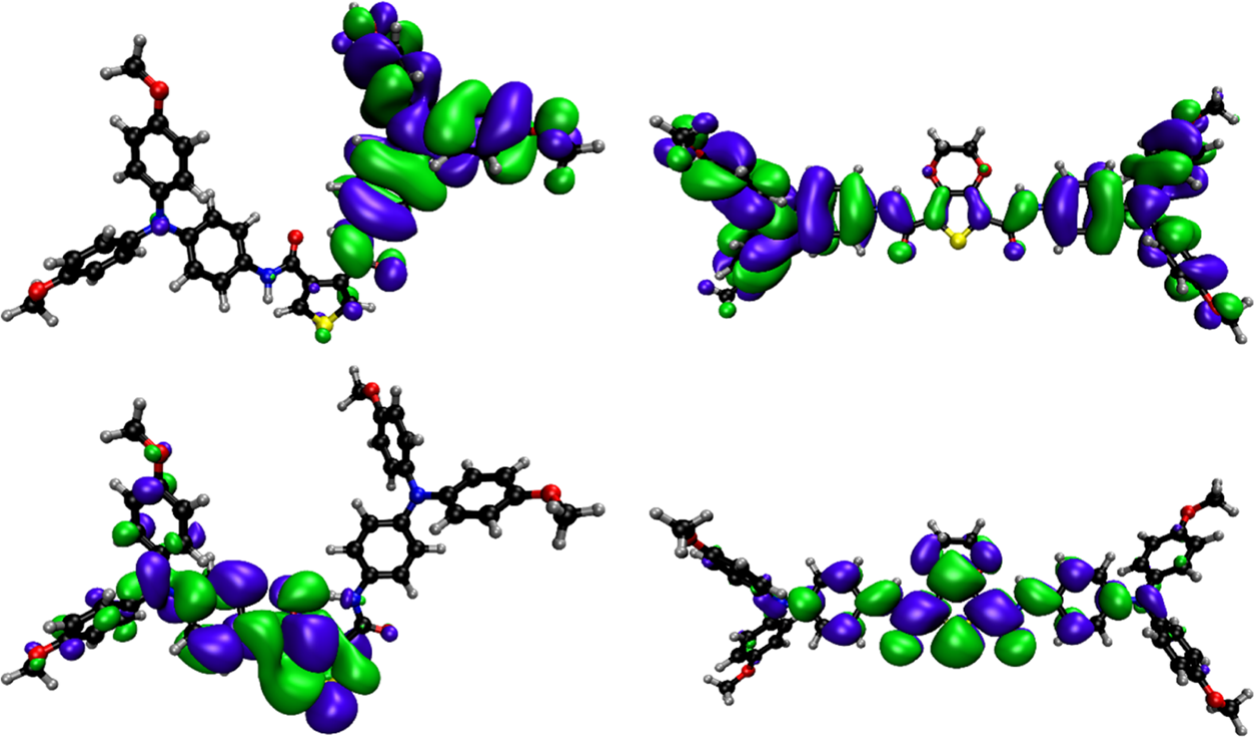
Frontier orbital distributions of TPABT (top left: HOMO; bottom left LUMO) and EDOT-Amide-TPA (top right: HOMO; bottom right: LUMO) in dichloromethane, from DFT (PBE0/def2-SV(P)).

To gain further insight into the structural and electronic properties of the amines, DFT calculations of the molecules were performed at the PBE0/def2-SV(P) level using ORCA (version 5.0.2).^25−30^ All calculations used the C-PCM implicit solvation model with the parameters of dichloromethane. Frequency calculations were performed on all energy-minimized geometries to ensure that minimum energy conformations were obtained. This shows (Figure 3) that, unlike EDOT-Amide-TPA, the HOMO of TPABT is localized on the TPA arm and the amide linker moiety. The orientation and distance of the carbonyl oxygen to the amide hydrogen on either side of the thiophene core point to the possibility of intramolecular hydrogen bonding (in agreement with the experimental crystal structure), resulting in asymmetry. This manifests in the destabilization and stabilization of the HOMO – 1 and LUMO + 1 energies, respectively. The hole-hopping distance may be approximated by the TPA N···N distances of HOMO sites on two neighboring molecules. The TPABT-CB crystal structure reveals the shortest hopping distance, 8.9 Å. The equivalent distances in TPABT2-CHCl_3_ and TPABT-THF are even shorter at 8.6 and 8.0 Å, respectively.

The HOMO energies obtained for single molecules in a vacuum are likely to vary from those in the film. Some of the effects of neighboring molecules can be approximated by implementing the C-PCM, with the parameters set to dichloromethane (κ = 9.08).^25−27^ It has previously been shown that a correction factor can be applied to theoretical HOMO energies calculated in dichloromethane, giving results that match closely with those from cyclic voltammetry experiments.^28,29^ Using a procedure reported by Chi et al.,^30^ we obtained a correction of −0.206 eV for the HOMO energies of a range of TPA- and DPA-based molecules in dichloromethane (Table 1 and Figure S4).

**Table 1:** Calculated Molecular Energy Levels for the HTMs Using DFT

frontier orbital energies (eV)						
compound	HOMO_DFT, vac_^a^	HOMO_DFT, DCM_^b^	HOMO_calc_^c^	LUMO_DFT, vacuum_^a^	LUMO_DFT, DCM_^b^	dipole_DFT, DCM_^b^ (debye)
TPABT	–4.87	–5.03	–5.23	–1.16	–1.34	10.6
EDOT-Amide-TPA^13,21,24,31^	–4.87	–5.09	–5.29	–1.82	–2.01	13.0
spiro-OMeTAD^13,21,24,31^	–4.60	–4.91	–5.12	–0.75	–1.09	3.26

a PBE0/def2-SV(P).

b PBE0/def2-SV(P) with C-PCM(CH_2_Cl_2_) implemented.

c based on HOMO_DFT,DCM_ with correction factor (−0.206 eV) applied.

The calculated HOMO energy of TPABT (−5.23 eV) was found to be close to the experimentally determined value (−5.29 eV) and, like EDOT-Amide-TPA, is suitably higher than the perovskite valence band (−5.43 eV). Interestingly, TPABT displays a high dipole moment, although lower than that of EDOT-Amide-TPA. While the high dipole of EDOT-Amide-TPA was previously offered as a possible explanation for its high mobility, by promoting close molecular packing in the solid film, recent work in our group has shown this to be unlikely.^32^ Indeed, a higher dipole generally correlates with a lower mobility,^33,34^ as the dipole–dipole interactions give rise to large energy barriers that restrict the mobility of the charge particles to smaller and smaller regions of the film; therefore, EDOT-Amide-TPA performs well despite this. Consequently, the higher mobility of TPABT compared with EDOT-Amide-TPA may be partly attributable to its lower dipole moment.

### Optical and Electrochemical Characterization

**Figure 4:**
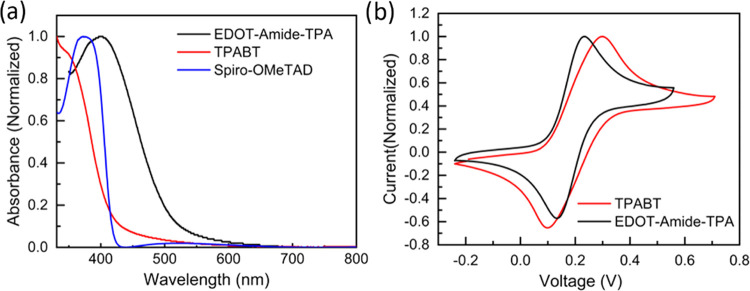
(a) Normalized UV–visible absorption spectra of TPABT (50 nm thickness), EDOT-Amide-TPA (45 nm), and Spiro-OMeTAD (200 nm) as a thin film that was applied by spin coating under the same conditions as the solar cells (below). (b) Normalized cyclic voltammogram of TPABT and EDOT-Amide-TPA measured in anhydrous dichloromethane under N_2_ with 0.1 M TBAPF_6_.

To maximize light-harvesting by the perovskite layer, minimize parasitic absorption losses, and enhance the current in solar cells, light absorption by the HTM in the range of visible light absorption from 400 to 800 nm should be minimized. Thus, the optical properties of TPABT, EDOT-Amide-TPA, and Spiro-OMeTAD films were investigated by using UV–visible absorption spectroscopy. The maximum absorption wavelengths of the HTMs are listed in Table 2. The absorption spectra of all three HTM thin films are displayed in Figures 4a and S5. The pristine Spiro-OMeTAD exhibits strong absorption in the UV region, with a peak maximum at 374 nm, which is consistent with data published previously by Wang et al.^36^ In contrast, the absorbance of pristine EDOT-Amide-TPA and TPABT films were very low in the UV region.^37^ We found this new material, TPABT, to be more transparent in the visible region compared to both Spiro-OMeTAD and EDOT-Amide-TPA (Figure S6), which will allow more photons to be absorbed by the perovskite layer and improve the PCE.

**Table 2:** Absorption Maxima of TPABT, EDOT-Amide-TPA, and Spiro-OMeTAD as Pristine Films and Doped with Added LiTFSI and the Redox Potentials of the HTMs in Dichloromethane

HTM	λ_max_ pristine (nm)	λ_max_ doped (nm)	*E*_g_^a^ (eV)	*E*_1/2_[HTM^+^|HTM] (*V* vs *F*_c_)	*E*_HOMO_^b^ (eV)	*E*_LUMO_^c^ (eV)
spiro-OMeTAD	374	376	2.70^10,35^	0.13	–5.20	–2.50
EDOT-Amide-TPA	408	430	2.73^13^	0.18	–5.25	–2.52
TPABT	333	332	2.70	0.22	–5.29	–2.594

a *E*_g_ is the HOMO and LUMO separation derived from the optical spectra.

b Calculated from *-*5.07 eV -*E*_1/2_[HTM^+^|HTM] + eV.

c Estimated from *E*_HOMO_ + *E*_g_.

On the addition of LiTFSI to TPABT, we observed a shift in the absorption maximum, indicating a change in the electronic properties of the film (Figure S7). This is consistent with previous research by Petrus et al.^13^ We also observed that the absorption maxima of Spiro-OMeTAD and EDOT-Amide-TPA decreased upon the addition of LiTFSI, compared to their pristine absorption. Petrus et al. previously demonstrated by FTIR spectroscopy that LiTFSI plays a role in doping the HTMs through the coordination of the Li^+^ ion to the amide bond, and this coordination leads to increased conjugation through the amide bond.^13^ This leads to an increase in carrier density, charge delocalization, and conductivity of the film.^38^

It is essential to have a good alignment of the HOMO energy level of the HTM with the valence band of the chosen perovskite to successfully extract holes and obtain a high open-circuit voltage (*V*_OC_). Hence, the electrochemical behavior of TPABT was investigated by cyclic voltammetry (CV), and the data are shown in Figure 4b and Table 2. We observed reversible oxidation starting at 0.17 V versus *F*_C_ and the redox potential of TPABT was determined to be 0.22 V vs *F*_c_, which is more positive than that for EDOT-amide-TPA (0.18 V vs *F*_c_). From these values plus the optical energy gap (*E*_g_), the HOMO energy was calculated to be −5.29 eV and the LUMO energy was estimated to be −2.59 eV. The energy level of the HOMO is in good agreement with the valence band of the perovskite (−5.23 eV)^13^ and is expected to result in minimal losses in *V*_OC_, while the high LUMO energy allows TPABT to function as an effective electron-blocking layer.

### Thermal Properties

**Figure 5:**
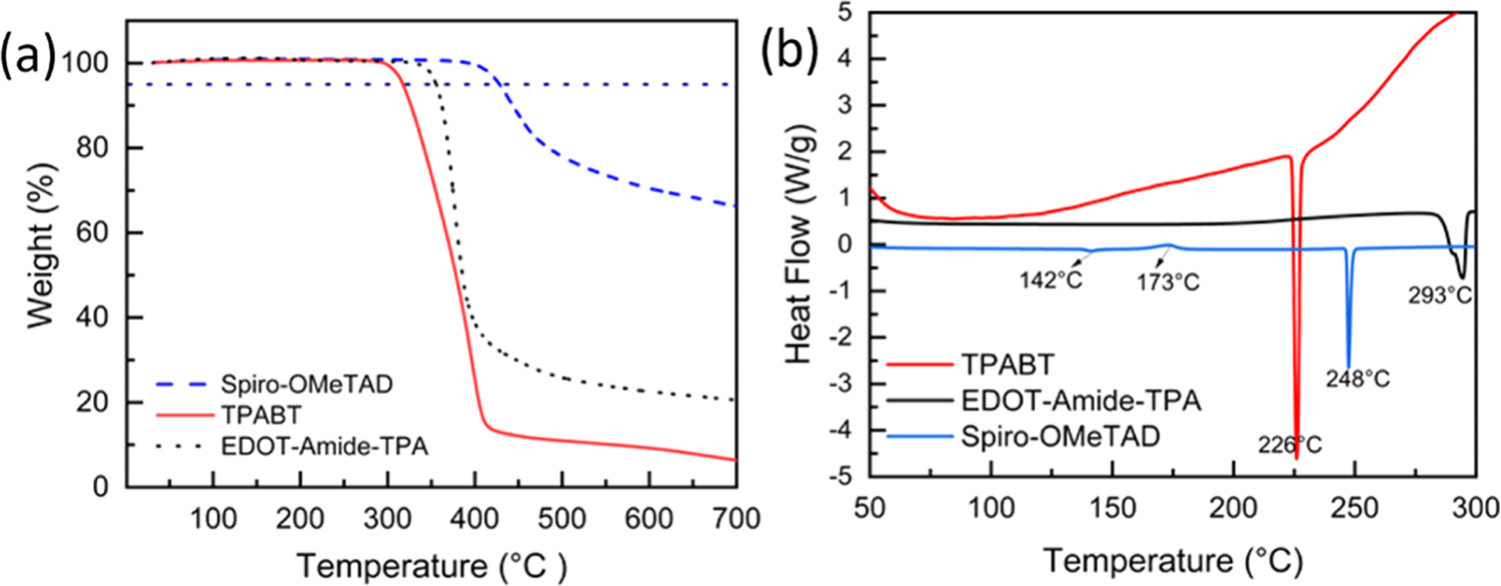
(a) Thermogravimetric analysis of pristine TPABT, EDOT-Amide-TPA, and Spiro-OMeTAD at a heating rate of 5 °C min^–1^ under N_2_ atmosphere. (b) Differential scanning calorimetry (first cycle of heating) for TPABT, EDOT-Amide-TPA, and Spiro-OMeTAD at the heating rate of 5 °C min^–1^.

One important advantage of aromatic amides is their outstanding thermal and chemical stability.^39^ This is important when it comes to photovoltaic devices as the HTM should not degrade under full operational conditions, which can reach over 80 °C.^13,40^ The thermal properties of TPABT were investigated by using thermogravimetric analysis (TGA) and differential scanning calorimetry (DSC) measurements compared with EDOT-Amide-TPA and Spiro-OMeTAD. The results of TGA for HTMs, (Figure 5a) show that the TPABT behaves similarly to EDOT-Amide-TPA and Spiro-OMeTAD below 300 °C with mass loss of less than 1%. The degradation temperature was 300 °C for TPABT and 347 °C for EDOT-Amide-TPA, which is very close to the value published by Petrus et al., while the degradation temperature of Spiro-OMeTAD was 408 °C, also consistent with a previous study.^13^ However, after doping the TPABT and Spiro-OMeTAD by LiTFSI and *t*BP, the degradation temperature of TPABT shifted from 300 to 350 °C, while the spiro-OMeAD started degrading from 250 °C, indicating that TPABT is more thermally stable than Spiro-OMeTAD (Figure S10). The DSC analysis can expose the polymorph character of HTMs, and the melting point also indicates the stability of the materials. In Figure 5b, the DSC of Spiro-OMeTAD in the first cycle of heating shows the glass transition *T*_g_ at 142 °C, a crystallization temperature *T*_C_ of 173 °C, and the melting point *T*_m_ at 248 °C. In the second cycle of heating, the *T*_g_ had shifted to 124 °C, while the *T*_m_ and *T*_C_ were not observed (Figure S13), and those values obtained match well with the values reported in the literature.^41^ By comparison, the first cycle of heating TPABT and EDOT-Amide-TPA showed the *T*_m_ at 226 and 293 °C, respectively. The *T*_m_ appeared at the same temperature in the second wave of heating for EDOT-Amide-TPA; however, the TPABT was amorphous in the second cycle (Figures S11 and S12). All thermal transitions are well above the operating temperature of photovoltaic devices (Table S6).

### Charge Transport Measurements

**Figure 6:**
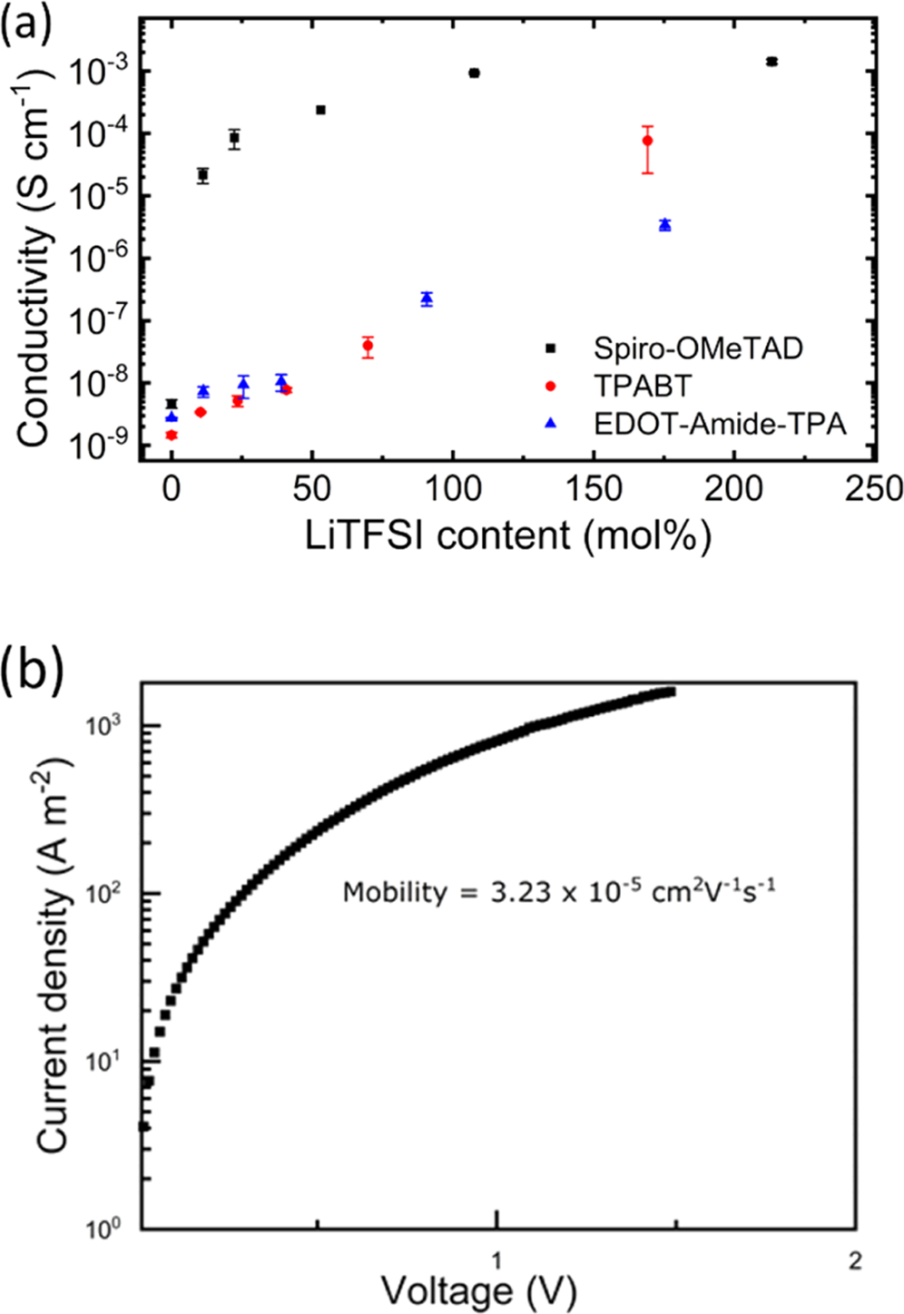
(a) Conductivity of the film vs concentration of LiTFSI. 20% error is added to account for the deviation in film thickness across the samples (± 10 nm). (b) *J*–*V* measurements of hole-only devices.

To gain a better understanding of the charge transport, we measured the hole mobilities of the HTMs in devices using the following architecture: ITO/PEDOT:PSS/TPABT/Au; the results are shown in Figures 6b and S14 and summarized in Table S7. The estimated hole mobility in TPABT upon the addition of LiTFSI was 3.23 × 10^–5^ cm^2^ V^–1^ s^–1^. The hole mobility in Spiro-OMeTAD and EDOT-Amide-TPA have been reported to reach of 5.3 × 10^–4^ and 2.1 × 10^–4^ cm^2^ V^–1^ s^–1^, respectively.^13^ Upon the addition of LiTFSI to the HTM, a significant increase in mobility was observed, which can be ascribed to the reduction in the barrier height for charge hopping between TPA units.

In addition to the mobility of charge carriers, high conductivity is essential for HTMs. Thus, we performed in-plane conductivity measurements for thin films of the HTMs with LiTFSI as an additive and the results are shown in Figure 6a. AFM images of the thin films of the HTMs on glass are shown in (Figures S15–S17). With an increase in the concentration of LiTFSI in the HTM solution, the conductivity of the TPABT films sharply increases, reaching a maximum value of 7.63 × 10^–5^ S cm^–1^ with 170 mol % LiTFSI. Lower conductivity was recorded for EDOT-Amide-TPA (3.40 × 10^–6^ S cm^–1^) with the addition of 175% LiTFSI. The higher conductivity value for TBABT compared to EDOT-Amide-TPA might arise from the 3D assembly of this material (Figure 2), and possibly an increase in the oxidation rate (Figure S7), which leads to a rise in conductivity.

### Device Characterization

**Figure 7:**
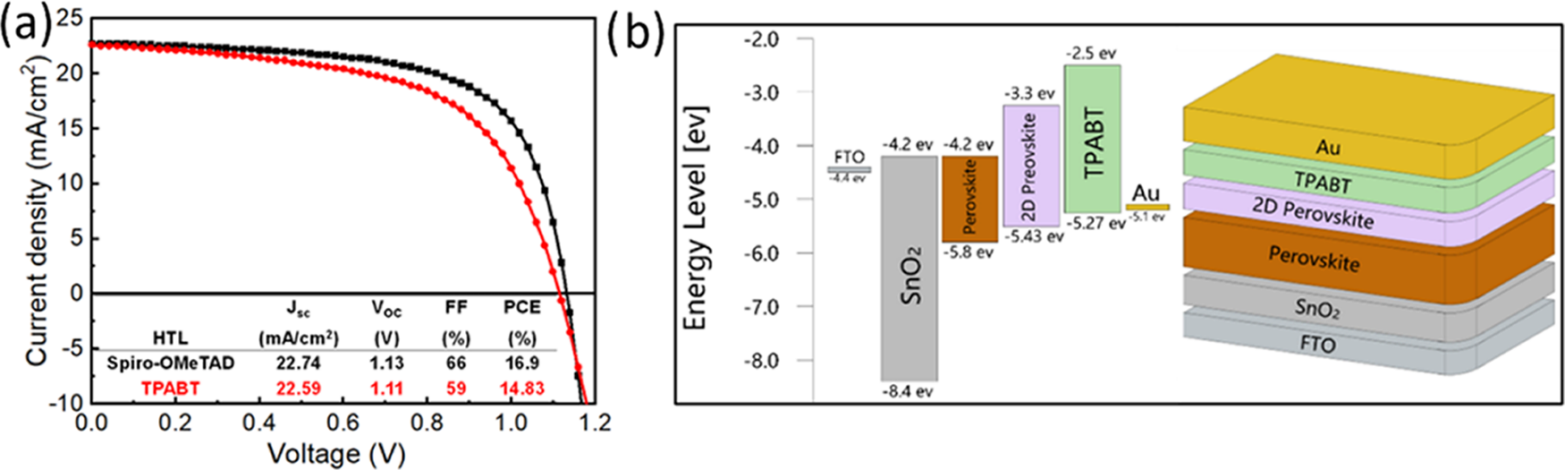
(a) *J*–*V* curves collected under AM 1.5 simulated sunlight of the champion device comprising TPABT and Spiro-OMeTAD in combination with FAMACs perovskite on SnO_2_. (b) Energy level diagram of TPABT with perovskite and ETL.

Solar cell devices were prepared to investigate the appropriateness of TPABT as an HTM in PSCs, and the results were compared with Spiro-OMeTAD. The TPABT deposition parameters were optimized with MAPbI_3_. Then, solar cells were fabricated with a state-of-the-art perovskite composition, as the mixture of halides and cations has been reported to outperform MAPbI_3_ when applied in solar cells.^7^ The planar configuration device was adopted with (FTO/SnO_2_/Cs_0.05_(FA_0.85_MA_0.15_)0.95Pb(I_3_Cl_2_)/2D perovskite/HTM/Au), as shown in Figure 7b. To enhance the conductivity and mobility of the HTM, TPABT was doped with tBP and LiTFSI. The solar cell devices were characterized by measuring the current density–voltage curve under standard AM 1.5 sun illumination (100 mW cm^–2^). The best devices are shown in Figure 7a, with 16.9 and 14.84% efficiency for Spiro-OMeTAD and TPABT, respectively. The slightly low efficiency of TPABT might be due to somewhat unfavorable energetics at the interface with the perovskite, which possibly hinders charge extraction rates or interfacial recombination. Moreover, the statistical analysis (Tables S9–S12) shows that the increases in power conversion efficiencies (PCE), open-circuit voltage (*V*_OC_), and short-circuit current density (*J*_SC_) are significant for TPABT after 2 days of oxidation (Figures S21–S24).

The stability of the devices was assessed by exposing HTM-coated perovskite films to >90% relative humidity over a period of 4.5 h. The results are shown in Figure S25. While both samples degraded over this period, at the end of the test, the dark color of the perovskite was still present for the TPABT-coated perovskite films. The Spiro-OMeTAD-coated samples were completely discolored, however. We propose that the improved stability of TPABT is related to the presence of the amide bonds, as the interactions between Li^+^ ions and the amide bonds facilitate a homogeneous distribution of the ions throughout the HTM and minimize their propagation. Moreover, this strong intermolecular interaction helps with film formation, as shown in AFM images in Figure S19, which in turn reduces the number of pinholes in the film. By comparison, Spiro-OMeTAD tends to form nanopores as well as larger pinholes (Figure S20), which leave some of the perovskite surface exposed, and therefore degradation is catalyzed at these sites. Taking both together, an increase in device stability similar to that observed for the parent material EDOT-Amide-TPA can be expected.^13^

## Conclusions

In summary, this work shows a way to design and produce a new low-cost hole transporting material (TPABT) that can outperform the previous material in the same generation, EDOT-Amide-TPA. The material displays favorable properties including molecular assembly, high transparency, and excellent charge transport. It can be synthesized through simple condensation chemistry, resulting in extremely low-cost materials, especially compared to Spiro-OMeTAD, which is produced at a very high cost due to the complicated synthesis and extensive purification. The HOMO of TPABT obtained from cyclic voltammetry and DFT calculations indicates a good alignment with the valence band of perovskite, suggesting hole selectivity and the potential for good performance in solar cell devices. Conductivity measurements show that the addition of LiTFSI as an oxidant results in a major increase of the conductivity reaching up to ∼10^–4^ S cm^–1^ upon addition of 175% mol LiTFSI and a hole mobility of ∼3 × 10^–5^ cm^2^ V^–1^ s^–1^. Furthermore, we show that TPABT forms pinhole-free films that increase the moisture stability of the underlying perovskite layer. We note that although optimally doped Spiro-OMeTAD regularly delivers ∼10^–3^ S cm^–1^ under similar conditions, TPABT can be deposited forming thinner, pinhole-free films which mitigate somewhat the effect of the lower conductivity value. When applied to perovskite solar cells, TPABT delivers a good efficiency of ∼15% with *V*_oc_ 1.11 V and *J*_sc_ 22.59 mA cm^–2^. Further investigation of the performance of TPABT derivatives is underway; it is relatively easy to switch out the core and tune the TPA units to improve the film characteristics, conductivity and transparency, which could lead to potential further improvements in efficiency.

## Experimental Section

### Synthesis of TPABT

All chemicals were purchased from commercial sources and used as received unless otherwise stated. Solvents for spectroscopic studies were of the highest purity available. The 4-amino-4′,4′′-dimethoxytriphenylamine was prepared using a previously reported method.^19^

### Synthesis of 3,4-Ethylenedioxythiophene-2,5-dicarbonyl Chloride

2.8 mmol of thiophene-3,4-dicarboxylic acid was dissolved in 40 mL of THF and 0.1 equiv of DMF was added. Then, 2.25 equiv of SOCl_2_ was added dropwise, resulting in a color change to yellow. After heating the solution for 2 h at 80 °C, the solution was allowed to cool to R.T. The solvents were removed under vacuum, resulting in acyl chloride in a quantitative yield as a yellow-brown solid, which was used in the next step without further purification.

### Synthesis of N^3^,N^4^-Bis(4-(bis(4-methoxyphenyl)amino)phenyl)thiophene-3,4-dicarboxamide TPABT

The total yield of the acyl chloride was dissolved in 40 mL of dry THF, and 2.25 equiv of 4-amino-4′,4′′-dimethoxytriphenylamine was added, followed by dropwise addition of 0.2 mL of triethylamine to the solution. The resulting dark red solution was heated to reflux for 2 h. The mixture was allowed to cool to R.T. overnight, reduced to 5 mL, and 10 mL of Et_2_O was added, resulting in a pale-yellow precipitate. The precipitate was filtered, washed twice with ethanol, and dried in vacuum to deliver the product as a pale beige solid with a yield of 70%. ^1^H NMR (400 MHz, DMSO-*d*_6_) δ (ppm): 10.33 (s, 2H, amide), 7.87 (s, 2H, thiophene), 7.56 (d, *J* = 8.9 Hz, 4H, phenyl), 7.04 (m, 8H, phenyl), 6.96 (d, *J* = 8.9 Hz, 4H, phenyl), 6.82 (m, 8H, phenyl), 3.80 (s, 12H, methoxy). ^13^C NMR (101 MHz, DMSO-*d*_6_) δ (ppm) = 13C NMR (101 MHz, CDCl3) δ 162.63, 155.81, 145.85, 141.27, 135.77, 132.60, 131.38, 126.24, 121.83, 121.59, 114.83, 55.66. FTIR: ν(cm^–1^): 3238 (vw), 3100 (vw), 3039 (w), 2948 (vw), 2834 (w), 1608 (s), 1557 (m), 1538 (m), 1501 (vs), 1463 (m), 1441 (w), 1429 (w), 1368 (w), 1310 (m), 1284 (m), 1240 (vs), 1188 (w), 1178 (m), 1104 (w), 1035 (s), 943 (vw), 911 (w), 892 (w), 569 (w), 824 (vs), 782 (m), 728 (s), 679 (w), 639 (w), 617 (w), 572 (m), 525 (m), 479 (w). HRMS (*m*/*z*): found [M + H] ^+^: 777.2737, calcd for C_46_H_40_N_4_O_6_SH: 777.2741.

### General Characterization Techniques

Electronic absorption spectra were recorded at RT by using a Shimadzu UV-1800 spectrophotometer. FT-IR spectrum was recorded with a PerkinElmer FT-IR Spectrum Two instrument. High-resolution electrospray ionization (ESI) mass spectrometry data were collected by the National Mass Spectrometry Facility (NMSF) in Swansea. ^1^H, ^13^C NMR spectra were recorded with a Jeol ECS 400 MHz instrument. Chemical shifts are referenced relative to the residual protonated solvent.

Crystal structure data for TPABT-CHCl_3_, TPABT-CB, and TPABT-THF was collected on an Xcalibur, Atlas, Gemini ultradiffractometer equipped with a fine-focus sealed X-ray tube (λ_Cu Kα_ = 1.54184 Å). Cell refinement, data collection, and data reduction were undertaken via software CrysAlisPro (Rigaku OD, 2015). For TPABT-CHCl_3_ and TPABT-CB, intensities were corrected for absorption via an analytical numeric absorption correction method using a multifaceted crystal model based on expressions derived by R.C. Clark & J.S. Reid.^42^ For TPABT-THF, an empirical absorption correction using spherical harmonics was implemented in the SCALE3 ABSPACK scaling algorithm.

Data for TPABT-CHCl_3_ were collected at beamline I19 at Diamond Light Source (λ_Synchrotron_ = 0.6889 Å).^43,44^ These data were processed using APEX3 (Bruker (2015). APEX3. BrukerAXS, Inc., Madison, Wisconsin).

Using Olex2,^45^ the structure was solved using XT^46^ and refined by XL.^47^ All nonhydrogen atoms were refined anisotropically, and hydrogen atoms were positioned with idealized geometry, with the exception of those bound to heteroatoms which were located using peaks in the Fourier difference map. The displacement parameters of the hydrogen atoms were constrained using a riding model with *U*_(H)_ set to be an appropriate multiple of the *U*_eq_ value of the parent atom.

### Cyclic Voltammetry (CV)

Cyclic voltammetry experiments were performed using glassy carbon as a working electrode, the counter electrode was platinum wire, and an Ag/AgCl reference electrode with and without a ferrocene (Fc) internal reference. Experiments were performed in anhydrous and degassed dichloromethane solutions of the hole transporter, with 0.1 M tetrabutylammonium hexafluorophosphate (tBuNPF_6_) as electrolyte and a scan rate of 50 mV s^–1^. HOMO levels were calculated as stated in the literature with the formal potential of the Fc+/Fc redox positioned at −5.07 eV versus vacuum.

### Thermal Characterization

Thermogravimetric analysis (TGA) was performed using an STA 6000 (PerkinElmer) under a nitrogen atmosphere with a scan rate of 5 °C per minute. Differential scanning calorimetry (DSC) was performed under a nitrogen atmosphere using TA Instruments with a heating rate in the range of 5 °C per minute.

### Mobility Measurements

The hole mobility was evaluated according to the literature.^48^ A hole-only device with the ITO/PEDOT:PSS/HTM/Au structure was fabricated. Indium tin oxide (ITO)-coated glass substrates (Kintec, 15–20 Ω/m^2^) were etched and washed with soap, deionized water, and ethanol and dried with nitrogen. The remaining organic residues were removed with a digital UV Ozone cleaner (Nova scan) for 15 min. Then, a PEDOT: PSS layer 10 nm thick was deposited by spin coater at 6000 rpm on the substrate for 60 s, followed by annealing at 120 °C for 10 min. A TPABT solution was prepared at a concentration of 10 mg/mL in chlorobenzene, and chloroform (2:1) was applied by spin coater over the annealed PEDOT:PSS layer, followed by deposition of a 50 nm thick gold film as a counter electrode. The characteristics of the *J*–*V* devices were recorded using the Keithley system. Mobility was calculated by the SCLC method using the Mott–Gurney equation.

### Conductivity Measurements

Solutions of EDOT-Amide-TPA, TPABT, and Spiro-OMeTAD were prepared in differing proportions of chlorobenzene and chloroform to account for the differing solubilities of these materials in these organic solvents. The exact quantities used were as follows. Spiro-OMeTAD: 52 mg of spiro-OMeTAD in 2005.0 mg of chlorobenzene to give a 28.8 mg/mL solution; EDOT-Amide-TPA: 21.3 mg of EDOT-Amide-TPA in 1055 mg of chlorobenzene and 1112.5 mg of chloroform to give a 12.6 mg/mL solution; this solution was kept on a hot plate at 70 °C due to the poor solubility of EDOT-Amide-TPA in organic solvents; TPABT: 22.1 mg of TPABT in 1024.3 mg of chlorobenzene and 1025.7 mg of chloroform to give a 13.7 mg/mL solution. This solution was also heated to 70 °C to ensure full dissolution of the HTM. As the solution was seen to be stable when cooled back to room temperature, the vial was kept at ambient temperatures throughout the experiment.

The HTM solutions were incrementally doped by direct addition of LiTFSI from a solution in acetonitrile (202.5 mg in 745.6 mg of acetonitrile, 213.5 mg/mL). Doping was performed between spin-coating steps, and the mol % of dopant to HTM was calculated at each step accounting for the volume of solution consumed during spin-coating as well as the added volume from the dopant solution.

Thin films of all our materials were spin-coated onto patterned ITO substrates that allowed measuring conductivity across a channel of width 60 μm and channel length 37.2 cm. Films were also spin-coated onto glass slides from which the film thickness was extracted using a DekTak XT profilometer equipped with a stylus tip of radius 2 μm. All films were left in a dry oxygen atmosphere for lithium-mediated oxidation for 4 days prior to measurement. JV curves were recorded by pulsing voltages across a 30 V range centered around 0 V, under ambient conditions using a Keithley 2400 source meter. From these JV curves and our pattern parameters, the conductivity was calculated for each condition. The conductivity values shown in Figure 6a and the corresponding error bars show the average of conductivities from three slides per condition, and the standard deviations, respectively.

### Device Characterization

Perovskite photovoltaics were fabricated in a standard device structure, FTO/SnO_2_-nanoparticles (np)/FAMACs perovskite/HTM/Au. FTO glass substrates were patterned with a laser etch and cleaned using a cleaning solution of Hellmanex (2%) in deionized water, acetone, and ethanol, and then dried with nitrogen. The substrates were then treated with UV ozone for 15 min. For the electron transport layer (ETL), SnO_2_-np (Alfa) diluted with deionized water (1:1) was spin-coated onto FTO substrates at 2000 rpm for 30 s in the air and annealed at 150 °C for 30 min. The substrates were immediately transferred to deposit a perovskite and (HTL) in the glovebox. The perovskite solution was prepared according to the following procedure: PbI_2_ (1.575M), PbCl_2_ (0.05M), CsI (0.075M), FAI (1.21M), and MAI (0.21M) were dissolved in DMF/DMSO mixture (4:1) and left on a hot plate overnight at 70 °C in the glovebox. The next day, the solution was filtered before being used. The solution was spin-coated over SnO_2_ at 1000 rpm for 10 s and 4000 rpm for 30 s to deposit the perovskite film. 300 μL of ethyl acetate was dropped onto the spinning substrate at 15 s, and the substrate was immediately transferred to a hot plate at 100 °C for 30 min.

Hole transport layer and electrode deposition: (Spiro-OMeTAD) was prepared by dissolving 75 mg of spiro-OMeTAD (Sigma-Aldrich) with 10 mg of (Li-TFSI) salt, 13.5 mg of (CoTFSI) in 1 mL of anhydrous chlorobenzene, and 29 μL(*t*BP). 50 μL of the Spiro-OMeTAD solution filtering was statically dispensed onto the substrate and then spin-coated at 4000 rpm for 38 s, then annealed to the film at 70 °C for 30 min.

A solution of TPABT was prepared from 10 mg of TPABT in 1 mL of 2:1 v/v anhydrous chlorobenzene and chloroform along with 10 μL of *t*BP and 25 μL of LiTFSI salt in acetonitrile (170 mg/mL) in turn to increase HTM conductivity. The TPABT solution was heated for 10 min until the solution became completely transparent (≈80 °C), and then it was passed hot through a 0.45 μm polytetrafluoroethylene (PTFE) syringe filter. 60 μL of the TPABT solution was rapidly dispensed to the substrates while spinning at 1250 rpm for 40 s and 2000 rpm for 5 s. The substrates were left in a desiccator for 24 h for oxidation of the HTM before the 50 nm gold electrode was deposited by thermal evaporation in a vacuum of 10^–5^ Pa.

## Supporting Information Available

Details on the characterization of TPABT; NMR spectrum; crystal structure with different solvents; DFT calculations; UV–visible spectra; thermal properties; charge transport; AFM topography; and device characterization (PDF)
