# Supplementary material for: Improving the Conductivity of Amide-Based Small Molecules through Enhanced Molecular Packing and Their Application as Hole Transport Mediators in Perovskite Solar Cells
Source: ACS Appl Energy Mater. 2023 Nov 8;6(22):11573–82. doi: 10.1021/acsaem.3c01988 (PMC10685326; doi:10.1021/acsaem.3c01988)
Supplement: Supplementary file 1 — ae3c01988_si_001.pdf [file ae3c01988_si_001.pdf]

Supporting Information

Improving the Conductivity of Amide-based Small Molecules Through Enhanced Molecular Packing and Their Application as Hole Transport Mediators in Perovskite Solar Cells

Eman A. A. Alkhudhayr[a,c], Dumitru Sirbu[a], Miriam Fsadni[a], Benjamin Vella[b], Bening T. Muhammad[a], Paul G. Waddell[a], Michael R. Probert[a], Thomas J. Penfold[a*], Toby Hallam,[a] Elizabeth A. Gibson[a*], Pablo Docampo[b*]

[a] Energy Materials Laboratory, Chemistry, School of Natural and Environmental Sciences, Bedson Building, Newcastle University, Newcastle upon Tyne, NE1 7RU, UK.
Tom.Penfold@newcastle.ac.uk, Elizabeth.gibson@newcastle.ac.uk

[b] School of Chemistry, University of Glasgow, Glasgow, G128QQ, UK.
pablo.docampo@glasgow.ac.uk

[c] Department of Physics, College of Science, King Faisal University, 31982, Saudi Arabia.

Characterisation of TPABT

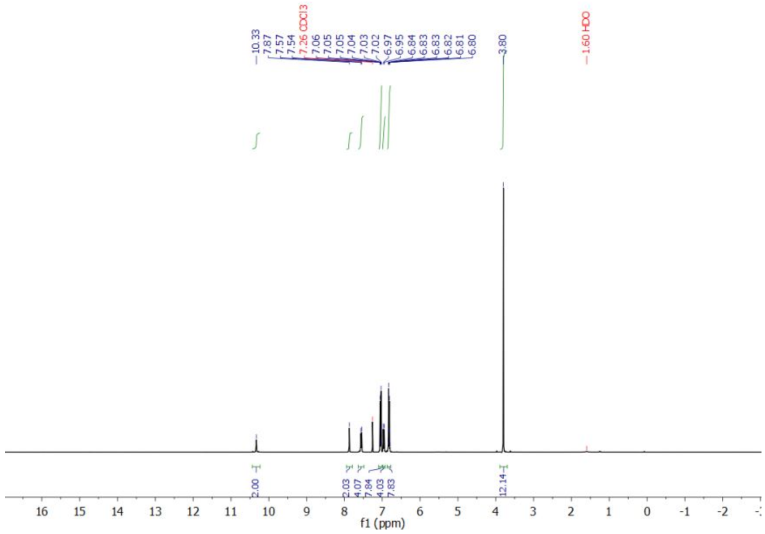

Figure S1. H NMR spectrum recorded at room temperature of TPABT in $CDCl_3$.

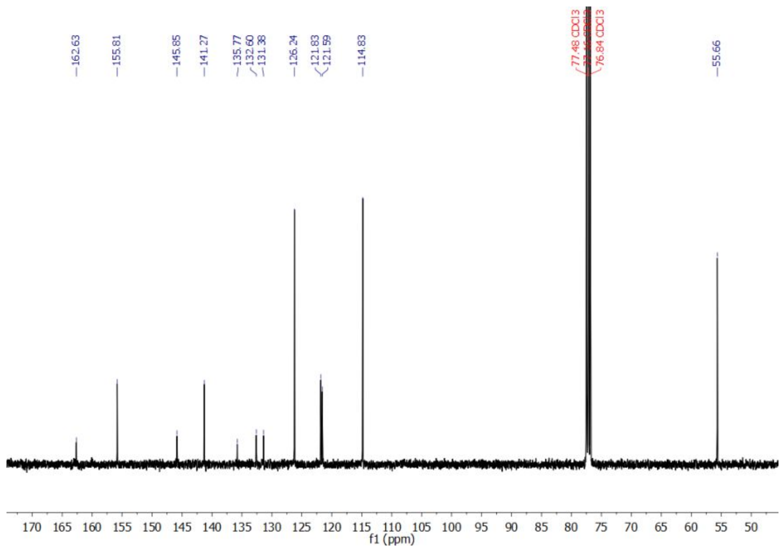

Figure S2. ^{13}C NMR spectrum recorded at R.T. of TPABT in $CDCl_3$.

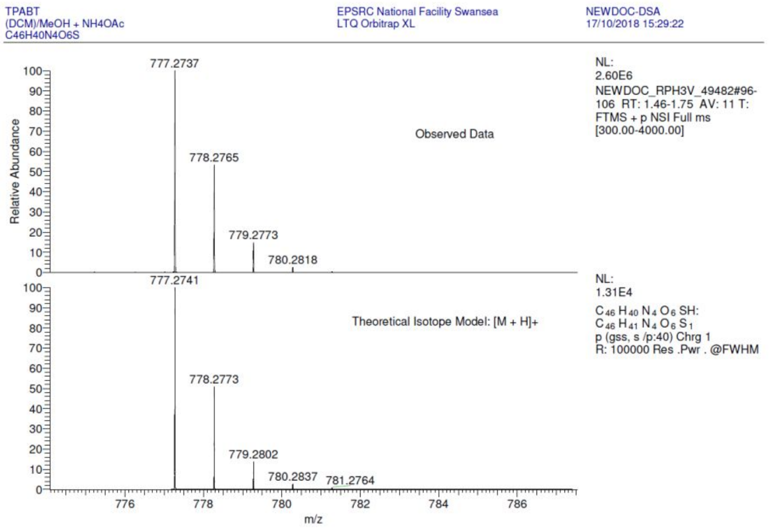

Figure S3. Observed and theoretical HRMS for [M+H]+ cation of TPABT.

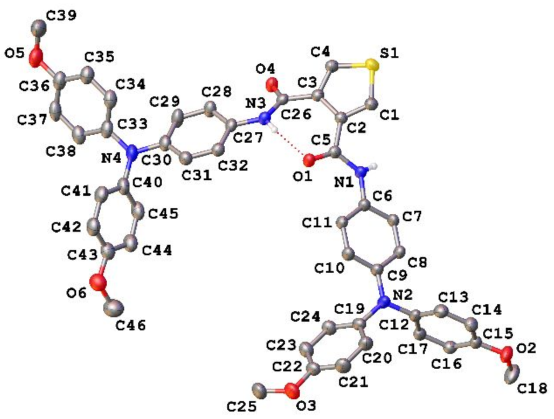

Table S1. Crystal data and structure refinement for the CB solvate of TPABT.

Identification code	TPABT-CB
Empirical formula	$C_{58}H_{50}Cl_2N_4O_6S$
Formula weight	1001.98
Temperature/K	150.0(2)
Crystal system	monoclinic
Space group	$P2_1/c$
a/Å	12.1297(3)
b/Å	14.8832(3)
c/Å	27.8998(7)
α/°	90
β/°	96.293(2)
γ/°	90
Volume/Å^3	5006.38(19)
Z	4
ρ_{calc}g/cm^3	1.329
μ/mm^{-1}	2.014
F(000)	2096.0
Crystal size/mm^3	0.22 × 0.05 × 0.05
Radiation	CuKα (λ = 1.54184)
2Θ range for data collection/°	7.332 to 133.852
Index ranges	$-14 \le h \le 14$, $-17 \le k \le 11$, $-33 \le l \le 32$
Reflections collected	36302
Independent reflections	8876 [R_{int} = 0.0653, R_{sigma} = 0.0490]
Data/restraints/parameters	8876/0/650
Goodness-of-fit on F^2	1.012
Final R indexes [I>=2σ (I)]	R_1 = 0.0527, wR_2 = 0.1327
Final R indexes [all data]	R_1 = 0.0809, wR_2 = 0.1522
Largest diff. peak/hole / e Å^{-3}	0.57/-0.57

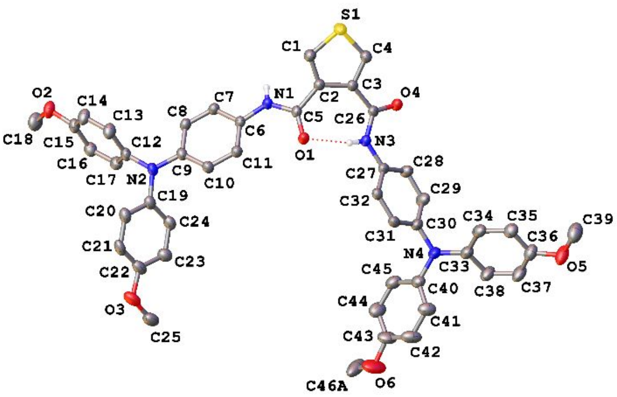

Table S2. Crystal data and structure refinement for the $CHCl_3$ solvate of TPABT.

Identification code	TPABT-$CHCl_3$
Empirical formula	$C_{47}H_{41}Cl_3N_4O_6S$
Formula weight	896.25
Temperature/K	150.0(2)
Crystal system	triclinic
Space group	P-1
a/Å	11.2176(3)
b/Å	15.2072(3)
c/Å	26.5071(6)
α/°	76.7998(19)
β/°	86.521(2)
γ/°	89.1071(19)
Volume/Å^3	4394.19(18)
Z	4
ρ_{calc}g/cm^3	1.355
μ/mm^{-1}	2.773
F(000)	1864.0
Crystal size/mm^3	0.3 × 0.21 × 0.08
Radiation	CuKα (λ = 1.54184)
2Θ range for data collection/°	7.534 to 133.832
Index ranges	$-13 \leq h \leq 13$, $-18 \leq k \leq 17$, $-31 \leq l \leq 31$
Reflections collected	61973
Independent reflections	15552 [R_{int} = 0.0396, R_{sigma} = 0.0298]
Data/restraints/parameters	15552/1081/1165
Goodness-of-fit on F^2	1.023
Final R indexes [I>=2σ (I)]	R_1 = 0.0504, wR_2 = 0.1350
Final R indexes [all data]	R_1 = 0.0625, wR_2 = 0.1462
Largest diff. peak/hole / e Å^{-3}	0.57/-0.63

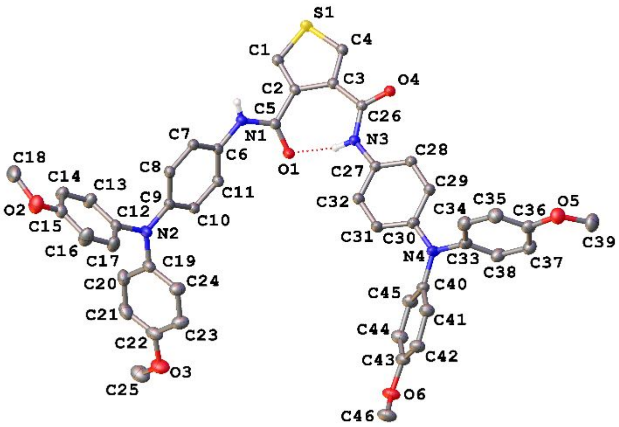

Table S3. Crystal data and structure refinement for the second polymorph of the $CHCl_3$ solvate of TPABT.

Identification code	TPABT2_$CHCl_3$
Empirical formula	$C_{47}H_{41}Cl_3N_4O_6S$
Formula weight	896.25
Temperature/K	100.0(2)
Crystal system	triclinic
Space group	P-1
a/Å	8.35220(10)
b/Å	14.70570(10)
c/Å	18.79250(10)
α/°	66.99
β/°	86.25
γ/°	88.95
Volume/Å^3	2119.93(3)
Z	2
ρ_{calc}g/cm^3	1.404
μ/mm^{-1}	0.301
F(000)	932.0
Crystal size/mm^3	0.236 × 0.049 × 0.023
Radiation	Synchrotron (λ = 0.6889)
2Θ range for data collection/°	2.286 to 55.48
Index ranges	$-11 \le h \le 11$, $-19 \le k \le 19$, $-25 \le l \le 25$
Reflections collected	32739
Independent reflections	10867 [R_{int} = 0.0675, R_{sigma} = 0.0687]
Data/restraints/parameters	10867/507/587
Goodness-of-fit on F^2	1.065
Final R indexes [I>=2σ (I)]	R_1 = 0.0557, wR_2 = 0.1708
Final R indexes [all data]	R_1 = 0.0613, wR_2 = 0.1767
Largest diff. peak/hole / e Å^{-3}	0.82/-0.80

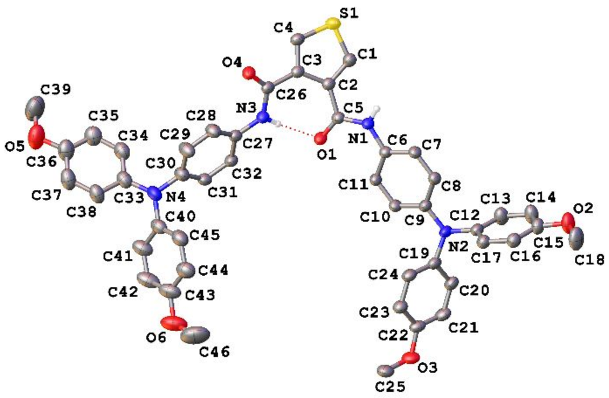

Table S4. Crystal data and structure refinement for the tetrahydrofuran (THF)solvate of TPABT.

Identification code	TPABT_THF
Empirical formula	$C_{108}H_{118}N_8O_{16}S_2$
Formula weight	1848.22
Temperature/K	150.0(2)
Crystal system	triclinic
Space group	P-1
a/Å	11.3945(2)
b/Å	15.1250(4)
c/Å	28.8132(7)
α/°	85.612(2)
β/°	81.4751(19)
γ/°	89.9926(18)
Volume/Å^3	4896.2(2)
Z	2
ρ_{calc}g/cm^3	1.254
μ/mm^{-1}	1.061
F(000)	1964.0
Crystal size/mm^3	0.27 × 0.21 × 0.07
Radiation	CuKα (λ = 1.54184)
2Θ range for data collection/°	7.846 to 133.802
Index ranges	$-13 \leq h \leq 13$, $-17 \leq k \leq 17$, $-34 \leq l \leq 32$
Reflections collected	68908
Independent reflections	17290 [R_{int} = 0.0488, R_{sigma} = 0.0375]
Data/restraints/parameters	17290/1527/1368
Goodness-of-fit on F^2	1.080
Final R indexes [I>=2σ (I)]	R_1 = 0.0773, wR_2 = 0.2073
Final R indexes [all data]	R_1 = 0.0929, wR_2 = 0.2176
Largest diff. peak/hole / e Å^{-3}	0.47/-0.41

Cost analysis

Table S5: The cost and quantities of the materials used to synthesise TPABT.

Chemical name	Weight(g)	Price of chemical ($/kg)	Material cost ($/g product)
Thiophene-diCOOH(1)	0.28	3,120.00	0.8736
DMF	0.01	5.09	0.0000509
SOCL2	0.44	27.67	0.0121748
THF	20.9	9.24	0.0193116
$TPA\text{-}NH_2$(3)	1.18	2,280.00	2.6904
Triethylamine	0.1	21.54	0.002154
THF	20.9	9.24	0.193116
EtOH	39.5	2.78	0.10981
MeOH	39.6	2.21	0.087516
Et2O	4.2	5.57	0.023394
Total			4.1853317

Density Functional Theory

HOMO energy correction:

Based on a previously reported method, theoretical HOMO levels of literature HTMs in dichloromethane were compared with their corresponding experimentally determined energies.[1,2] For a group of 15 HTMs[3–8] the DFT (PBE0/def2-SV(P) HOMO energies calculated in dichloromethane were plotted against their experimental values (Figure S4) and an excellent linear correlation was found (R^2 = 0.9548), with the relationship described by:

$$y = 0.9621x - 0.3959 \quad \textbf{(1)}$$

The molecules in this group consist of symmetrical HTMs with TPA (Docampo group amide, hydrazine linker- and azomenthine linker-based HTMs) and DPA side units, as well as spiro-OMeTAD. The root mean squared error (RMSE) between the theoretical and experimental data was found to be 0.206 eV.

Owing to the excellent linear correlation, applying a correction factor of -0.206 eV to the theoretical HOMO energies provided values in close agreement with experimental results, with a RMSE of 0.019 eV. When the initial group of HTMs was expanded to 23,[9–15] the correction factor of -0.206 eV was found to give a modest improvement in the theoretical HOMO energies, with a RMSE of 0.105 eV.

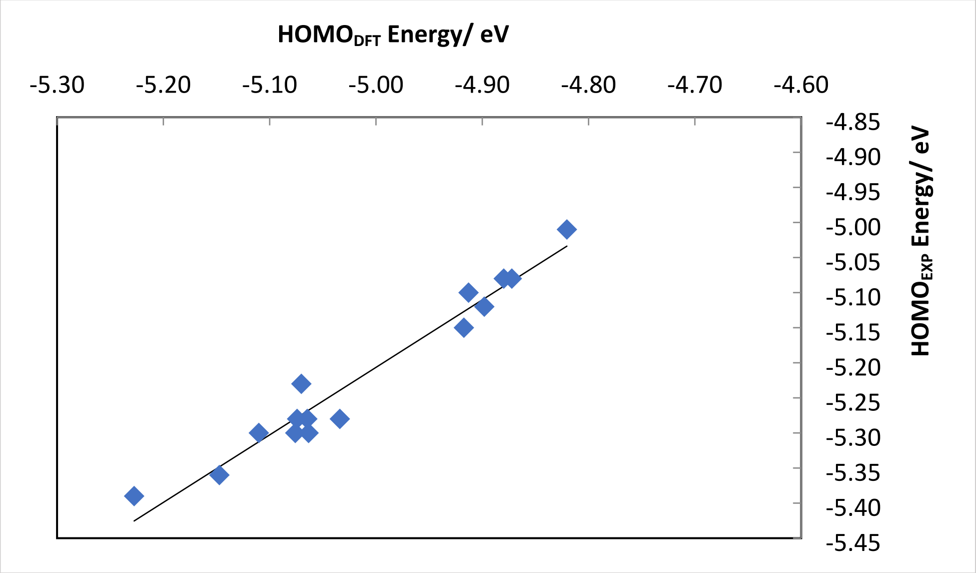

Figure S4. Experimental literature HOMO energies against their calculated values (DFT, PBE0/def2-SV(P), for a group of 15 HTMS.[3,4,13–15,5–12]

UV-Visible Absorption Spectroscopy

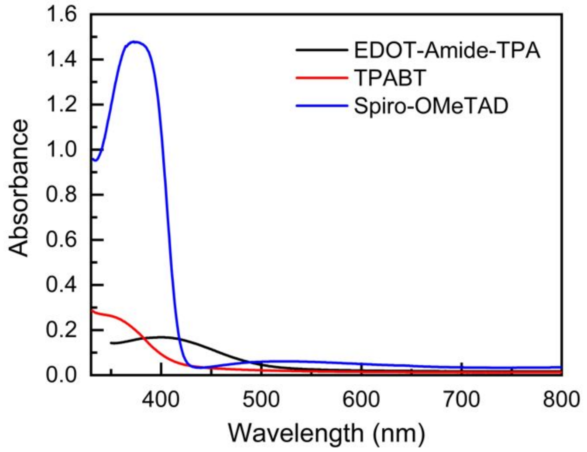

Figure S5. UV-vis absorption spectrum of TPABT (50 nm thickness), EDOT-Amide-TPA (45 nm), and Spiro-OMeTAD (200 nm) as thin film.

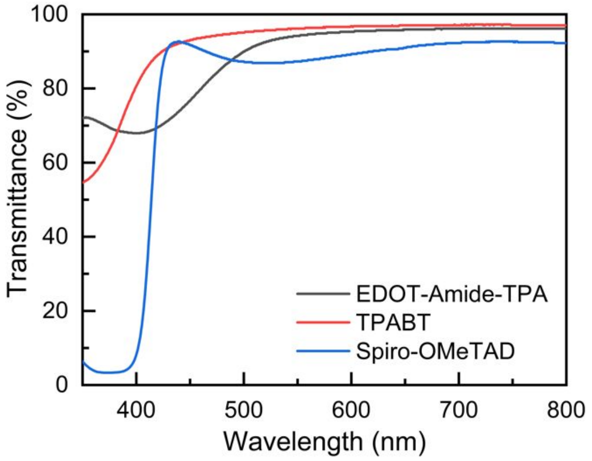

Figure S6. Transmittance of TPABT,EDOT-Amide-TPA, and Spiro-OMeTAD as thin film.

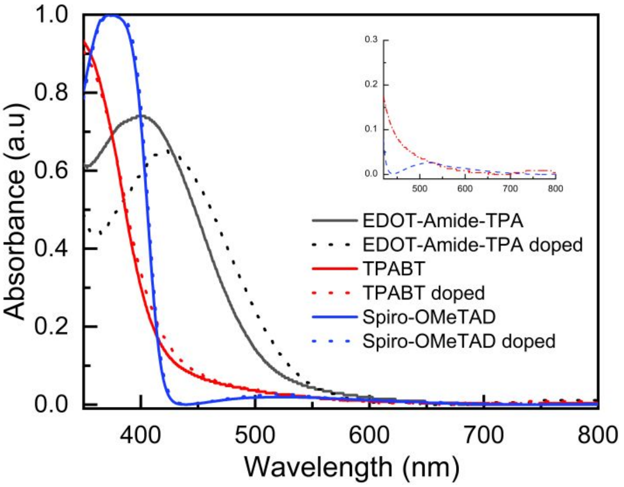

Figure S7. UV-vis absorption spectra of TPABT, EDOT-Amide-TPA, and Spiro-OMeTAD pristine and doped with Li-TFSI as a thin film.

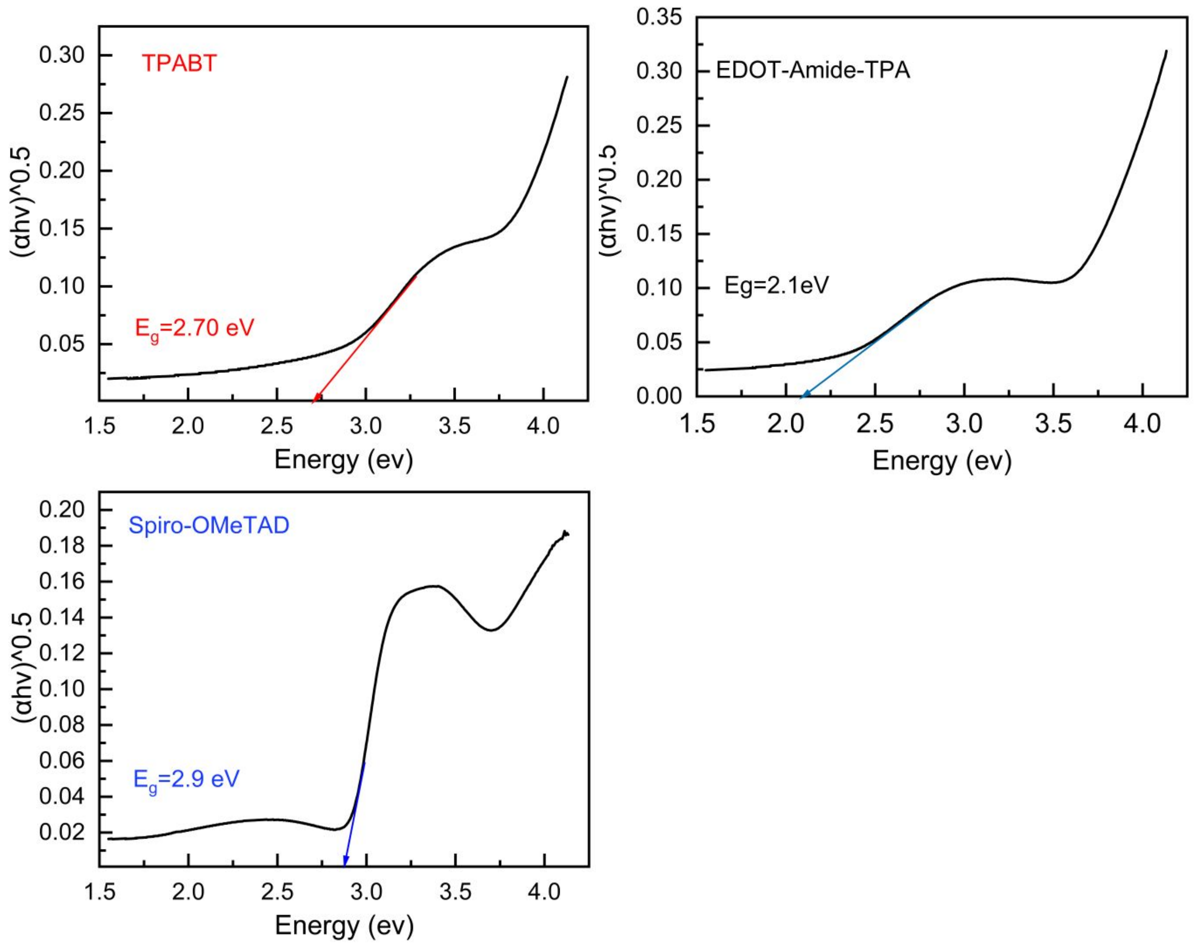

Figure S8. Band gap calculation of TPABT (50 nm thickness), EDOT-Amide-TPA (45 nm), and Spiro-OMeTAD (200 nm).

Cyclic Voltammetry

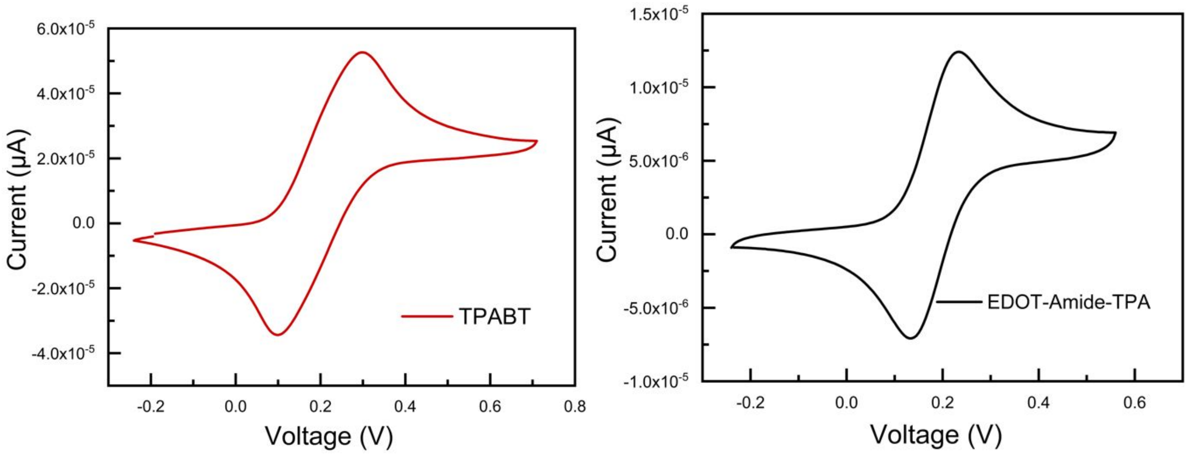

Figure S9. Cyclic voltammogram of TPABT and EDOT-Amide-TPA measured in anhydrous dichloromethane under N_2 with 0.1M tetrabutylammonium hexafluorophosphate $TBAPF_6$.

Thermal properties

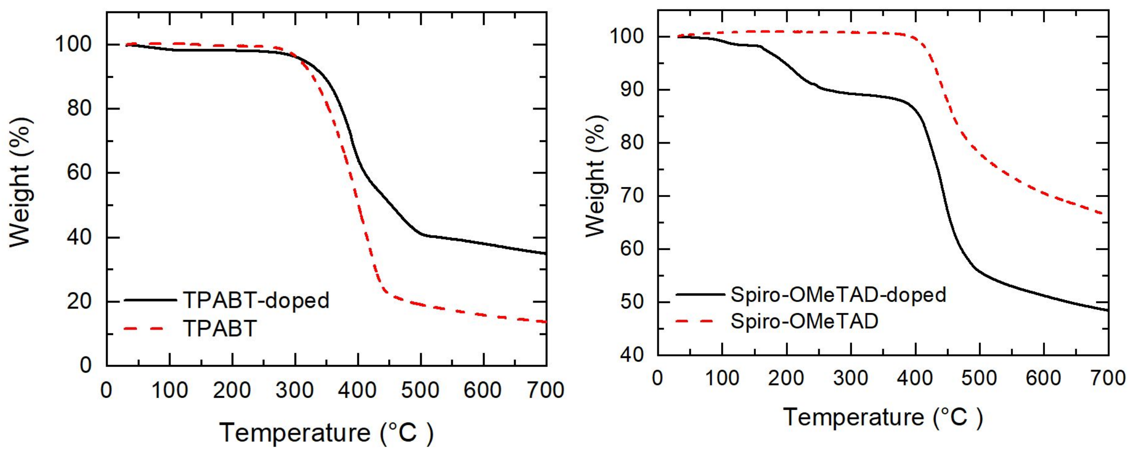

Figure S10. TGA thermograms of pristine and doped TPABT and Spiro-OMeTAD with a heating rate of 5 °C min^{-1} under nitrogen atmosphere.

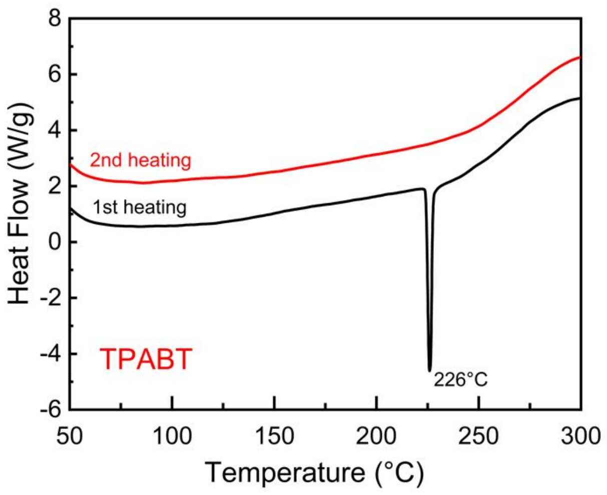

Figure S11. DSC first and second heating for TPABT, at the heating rate of 5° min^{-1}.

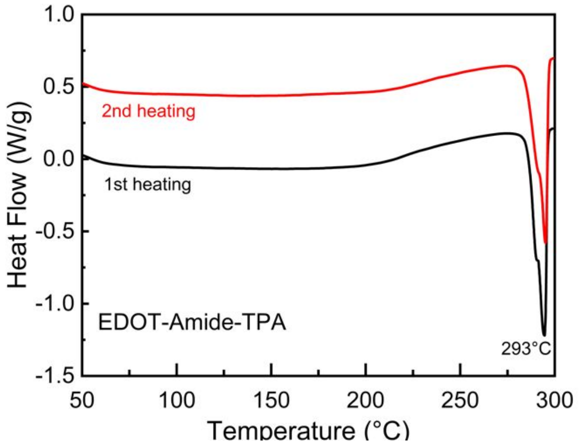

Figure S12. DSC first and second heating for EDOT-Amide-TPA, at the heating rate of 5° min^{-1}.

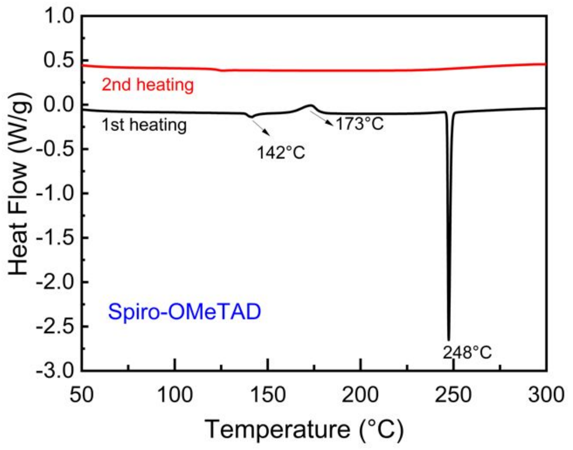

Figure S13. DSC first and second heating for Spiro-OMeTAD, at the heating rate of 5° min^{-1}.

Table S6. Comparison of the degradation, melting and glass transition temperature of EDOT-Amide, TPABT and Spiro-OMeTAD

HTL	T_m(°C)	T_d(°C)	T_g(°C)
Spiro-OMeTAD	248	408	142
EDOT-Amide-TPA	293	347	-
TPABT	226	300	-

Charge transport

Mobility

Due to the low mobility of charge carriers in organic semiconductors, the injected carrier builds up a space charge within the film. Assuming ohmic charge injection and trap free transport, the current will be space charge limited. In this regime, the current is only dependent on the mobility and not charge carrier density. The J-V curves were recorded, the charge carrier mobility was calculated using the Mott-Gurney equation ($J = \frac{9}{8}\mu\varepsilon_r\varepsilon_\circ\frac{V^2}{L^3}$) [16] (where J is the current density, μ is the hole mobility, ε_o is the vacuum permittivity, ε_r is the dielectric constant of the material, V is the applied bias and L is the thickness of the HTM).[17]

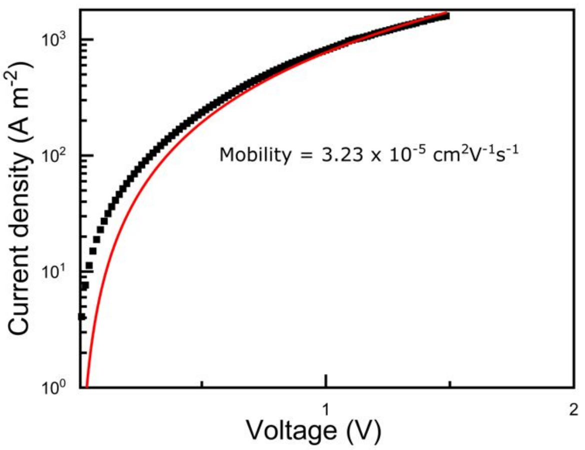

Figure S14. J-V measurements of hole only devices on a linear-log scale.

Table S7. Comparison of the mobility for Spiro-OMeTAD, EDOT-amide and TPABT, as pristine and with Li-TFSI doping

	TPABT	EDOT-Amide-TPA[3]	Spiro-OMeTAD[3]
Hole mobility ($cm^2\ V^{-1}\ s^{-1}$)	5.3×10^{-6}	3.9×10^{-5}	4×10^{-5}
Hole mobility doped ($cm^2\ V^{-1}\ s^{-1}$)	3.23×10^{-5}	2.1×10^{-4}	5.3×10^{-4}

Surface structure of HTM and HTM with perovskite films

Table S8. The experiment was done by using the XE-150 park system AFM to measure the thickness for TPABT, EDOT-Amide-TPA, and Spiro-OMeTAD at the same condition of solar cells.

Materials	TPABT	EDOT-Amide-TPA	Spiro-OMeTAD
Δy (nm)	50	45	200

TPABT

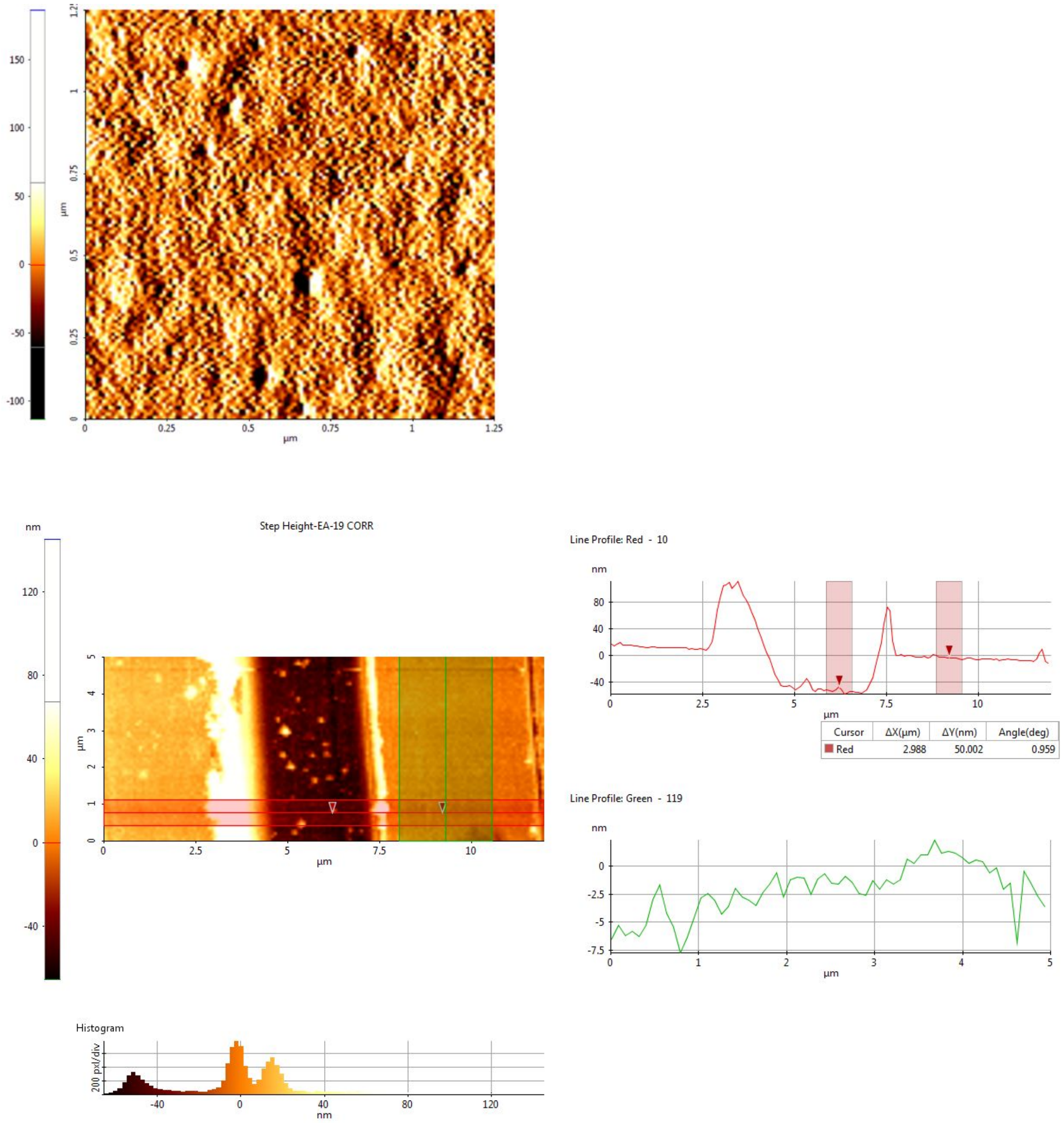

Statistics

Line	Min(nm)	Max(nm)	Mid(nm)	Mean(nm)	Rpv(nm)	Rq(nm)	Ra(nm)	Rz(nm)	Rsk	Rku
Red	-58.475	111.182	26.353	0.000	169.658	38.728	26.777	121.287	-0.871	4.077
Green	-7.729	2.305	-2.712	-2.185	10.034	2.275	1.805	7.188	0.460	2.648

Figure S15. NC mode AFM topography of TPABT (Rq=2.275 nm) film on glass substrate, scan area $5 \times 3\ \mu m$.

EDOT-Amide-TPA

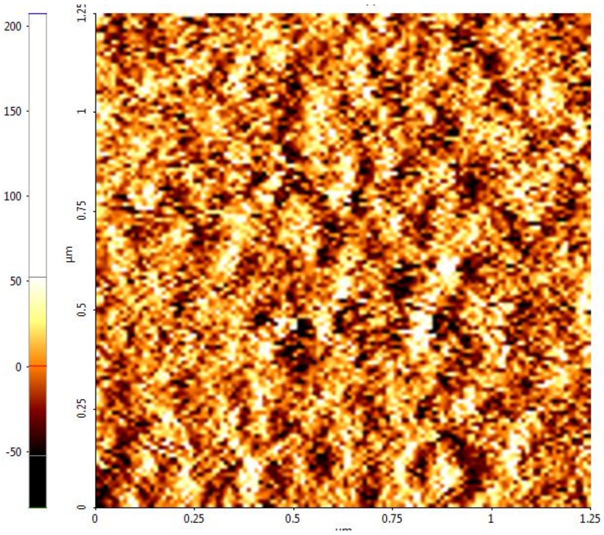

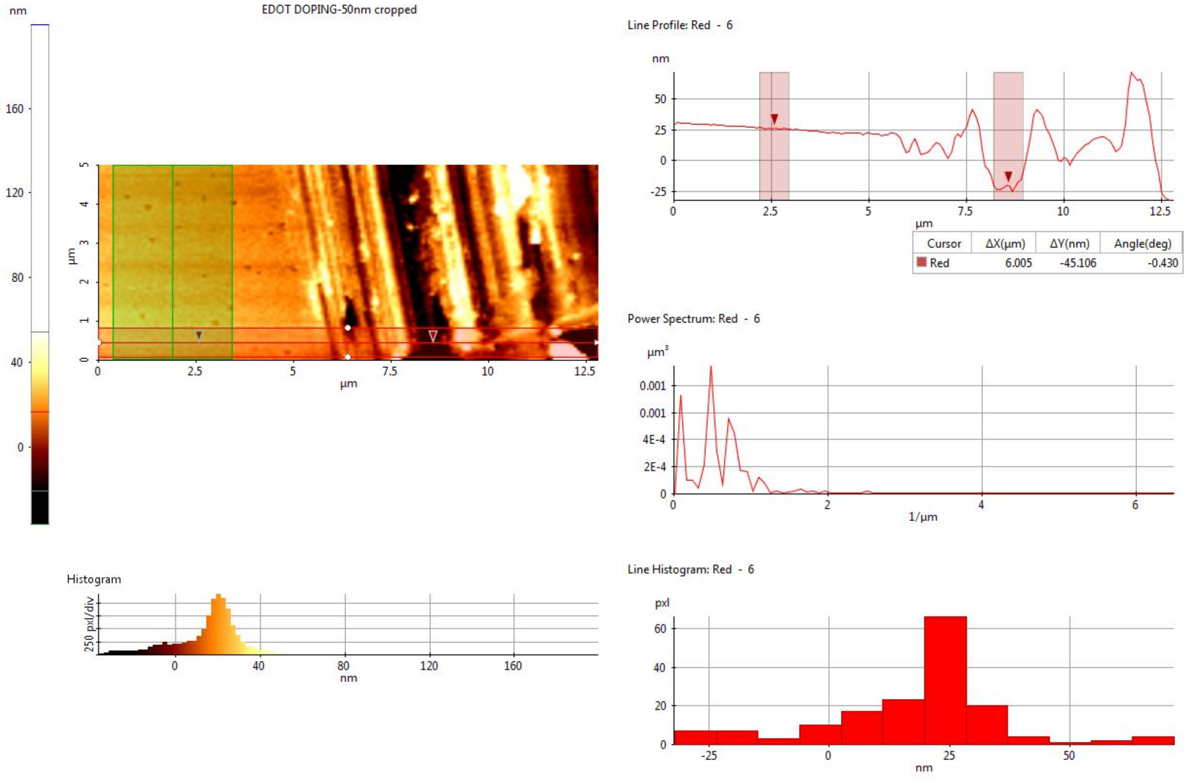

Statistics

Line	Min(nm)	Max(nm)	Mid(nm)	Mean(nm)	Rpv(nm)	Rq(nm)	Ra(nm)	Rz(nm)	Rsk	Rku
**Red**	**-32.316**	**71.532**	**19.608**	**17.543**	**103.848**	**18.313**	**13.236**	**60.192**	**0.395**	**4.413**
Green	17.252	29.926	23.589	22.808	12.674	3.270	2.659	9.622	-0.514	2.452

Figure S16. NC mode AFM topography of EDOT-Amide-TPA (Rq=3.2nm) film on glass substrate, scan area $5 \times 3\ \mu m$.

Spiro-OMeTAD

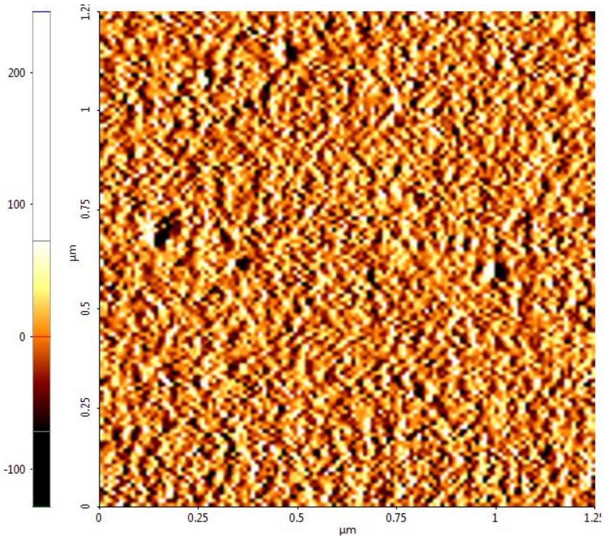

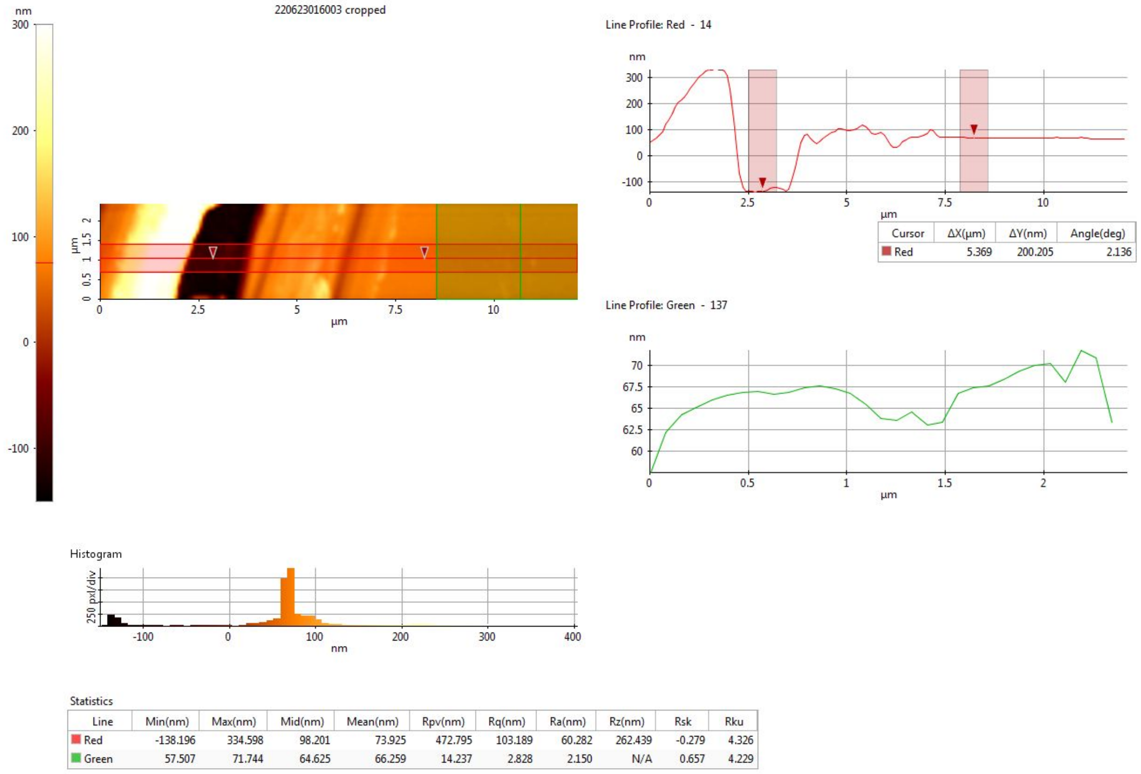

Figure S17. NC mode AFM topography of Spiro-OMeTAD (Rq=2.8 nm) film on glass substrate, scan area $5 \times 3\ \mu m$.

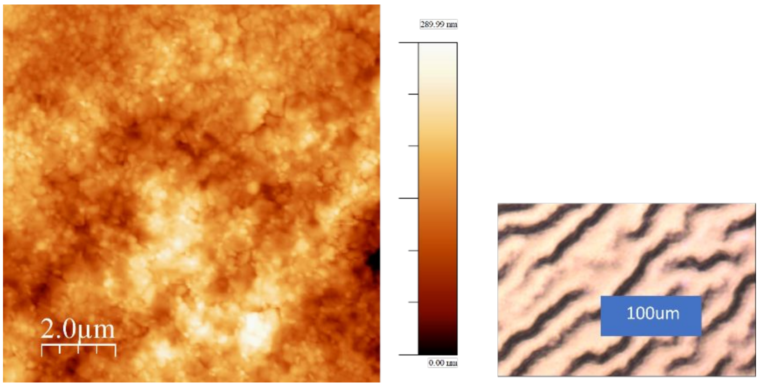

Figure S18. NC mode AFM topography of FAMACs perovskite (Rq = 39 nm).

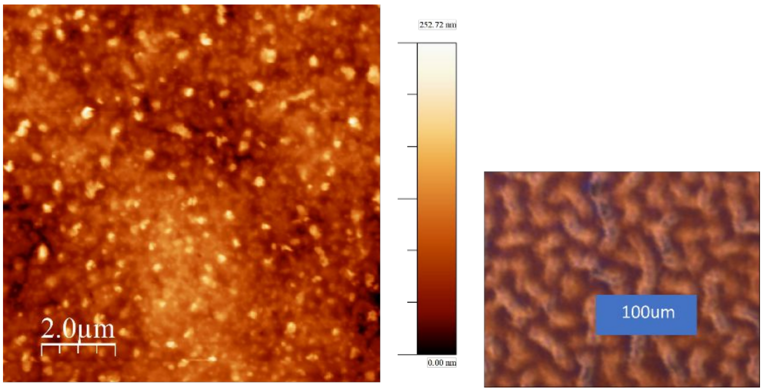

Figure S19. NC mode AFM topography of TPABT film upon the perovskite films within the device (Rq=29 nm), scan area 5×5 µm.

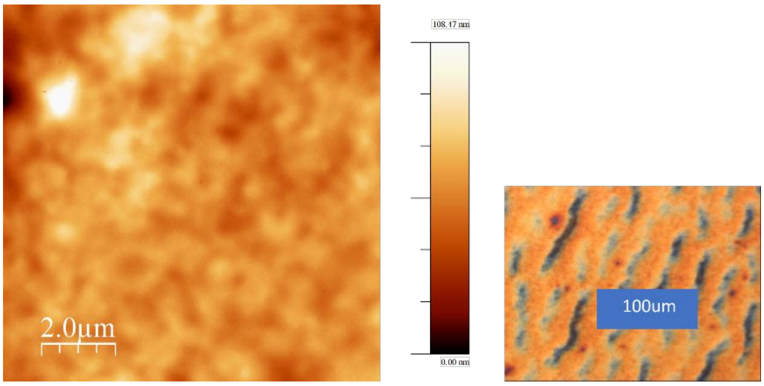

Figure S20. NC mode AFM topography of Spiro-OMeTAD film upon the perovskite films within the device (Rq= 10 nm), scan area 5×5 µm.

Device Characterization

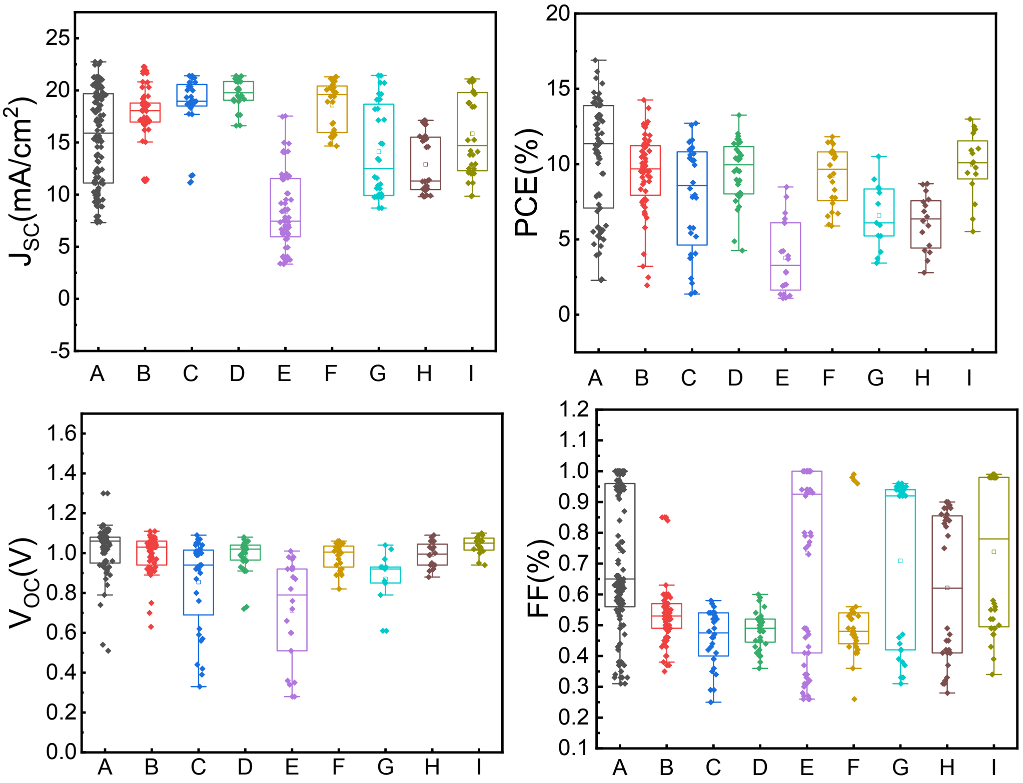

Figure S21. Photovoltaic performance FAMACs perovskite devices with SnO_2 as ETM. Box-plot of devices prepared in different parameters, A) Spiro-OMeTAD, B) TPABT doped with 25μL LiTFSi and 10 tBP, C) TPABT doped with 25μL LiTFSi and 12 tBP, D) TPABT doped with 25μL LiTFSi and 8 tBP, E) TPABT doped with 30μL LiTFSi and 10 tBP, F) TPABT doped with 30μL LiTFSi and 12 tBP, G) TPABT doped with 30μL LiTFSi and 8 tBP, H) TPABT doped with 35μL LiTFSi and 10 tBP, I) TPABT doped with 35μL LiTFSi and 12 tBP.

Table S9. The statistical analysis of the efficiency for devices (figure S20).

	Mean	Std. Deviation
A	10.4	3.8
B	9.4	2.6
C	7.8	3.6
D	9.5	2
E	3.8	2.3
F	9.2	1.9
G	6.6	2.21
H	6.2	1.9

I	9.9	2.2

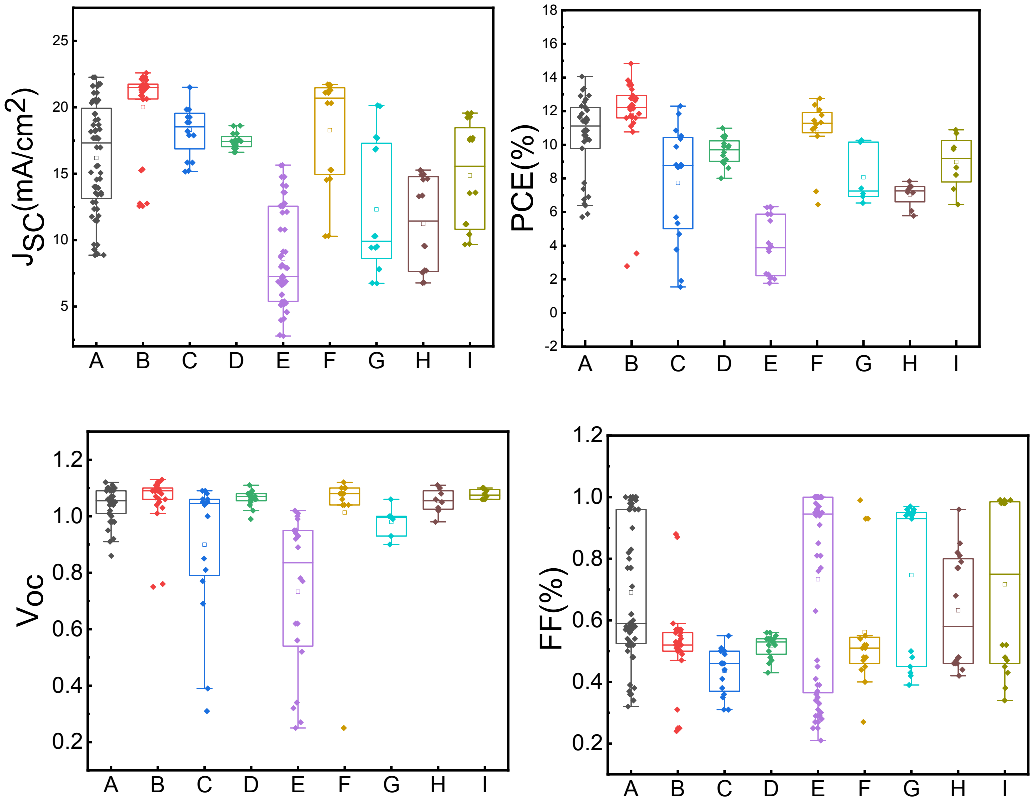

Figure S22. Re-measured photovoltaic devices after two days kept under N_2. Box-plot of devices prepared in different parameters, A) Spiro-OMeTAD, B) TPABT doped with 25μL LiTFSi and 10 tBP, C) TPABT doped with 25μL LiTFSi and 12 tBP, D) TPABT doped with 25μL LiTFSi and 8 tBP, E) TPABT doped with 30μL LiTFSi and 10 tBP, F) TPABT doped with 30μL LiTFSi and 12 tBP, G) TPABT doped with 30μL LiTFSi and 8 tBP, H) TPABT doped with 35μL LiTFSi and 10 tBP, I) TPABT doped with 35μL LiTFSi and 12 tBP.

Table 10. The statistical analysis of the efficiency for devices (figure S21).

	Mean	Std. Deviation
A	10.5	2.3
B	11.8	2.6
C	7.7	3.4
D	9.6	0.8
E	4	1.8
F	10.8	1.9
G	8	1.7

H	7	0.7
I	8.98	1.6

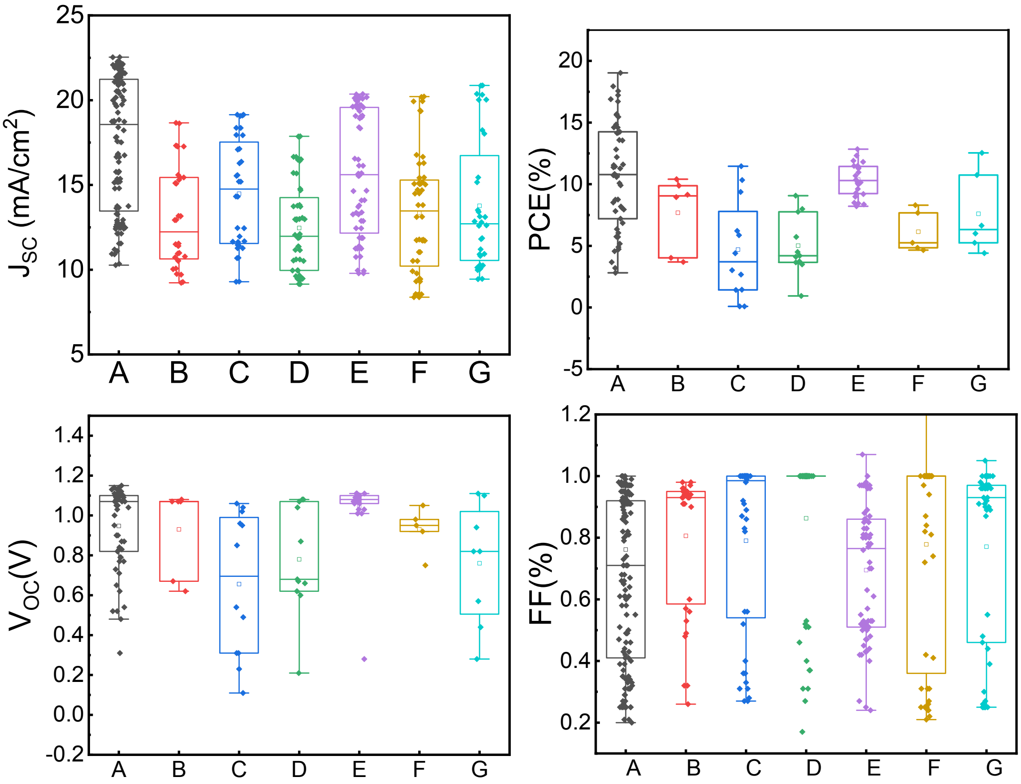

Figure S23. Photovoltaic performance FAMACs perovskite devices with SnO_2 as ETM. Box-plot of devices prepared in different parameters, A) Spiro-OMeTAD, B) TPABT doped with 25µL LiTFSi and 12 tBP, C) TPABT doped with 25µL LiTFSi and 12 tBP annealed at 60°C for 30 min, D) TPABT doped with 25µL LiTFSi and 12 tBP annealed at 70°C for 30 min, E) TPABT doped with 30µL LiTFSi and 12 tBP, F) TPABT doped with 30µL LiTFSi and 12 tBP annealed at 60°C for 30 min, G) TPABT doped with 30µL LiTFSi and 12 tBP annealed at70°C for 30 min.

Table S11. The statistical analysis of the efficiency for devices (figure S18).

	Mean	Std. Deviation
A	10.7	4.36
B	7.69	3.01
C	4.7	3.98
D	5.02	2.4
E	10.37	1.34
F	6.15	1.71
G	7.6	3.27

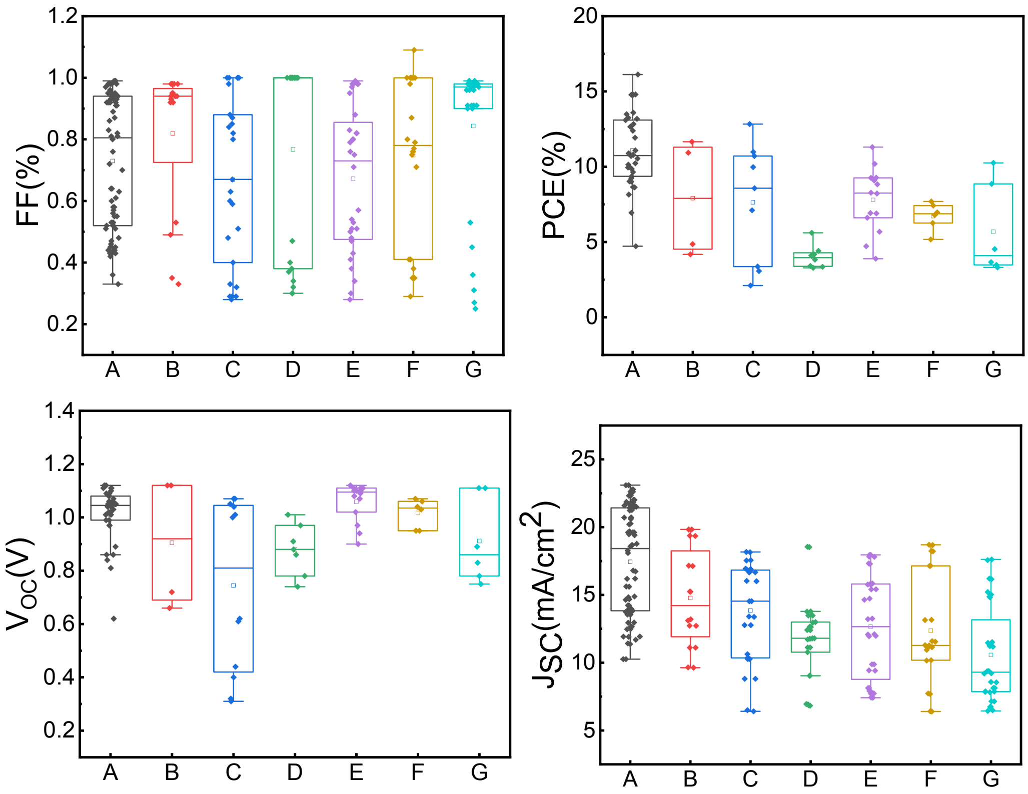

Figure S24. Re-measured photovoltaic devices after one day kept under N_2. Box-plot of devices prepared in different parameters, A) Spiro-OMeTAD, B) TPABT doped with 25µL LiTFSi and 12 tBP, C) TPABT doped with 25µL LiTFSi and 12 tBP annealed at 60°C for 30 min , D) TPABT doped with 25µL LiTFSi and 12 tBP annealed at 70°C for 30 min, E) TPABT doped with 30µL LiTFSi and 12 tBP, F) TPABT doped with 30µL LiTFSi and 12 tBP annealed at 60°C for 30 min, G) TPABT doped with 30µL LiTFSi and 12 tBP annealed at 70°C for 30 min.

Table S12. The statistical analysis of the efficiency for devices (figure S19).

	Mean	Std. Deviation
A	11.1	2.5
B	7.91	3.9
C	7.64	3.9
D	4.02	0.77
E	7.8	2.1
F	6.71	0.91
G	5.9	3.06

Moisture stability

Thin films of the two HTMs (doped Spiro-OMeTAD and doped TPABT) were coated on FAMAC perovskite as described for the solar cells. The edge of the samples was scraped off and covered with epoxy to prevent degradation from the side/rougher film area due to spin coating. The humidity test was carried out at with the devices placed in a box with relative humidity of >90% with the precaution not to let water to drip onto the samples.

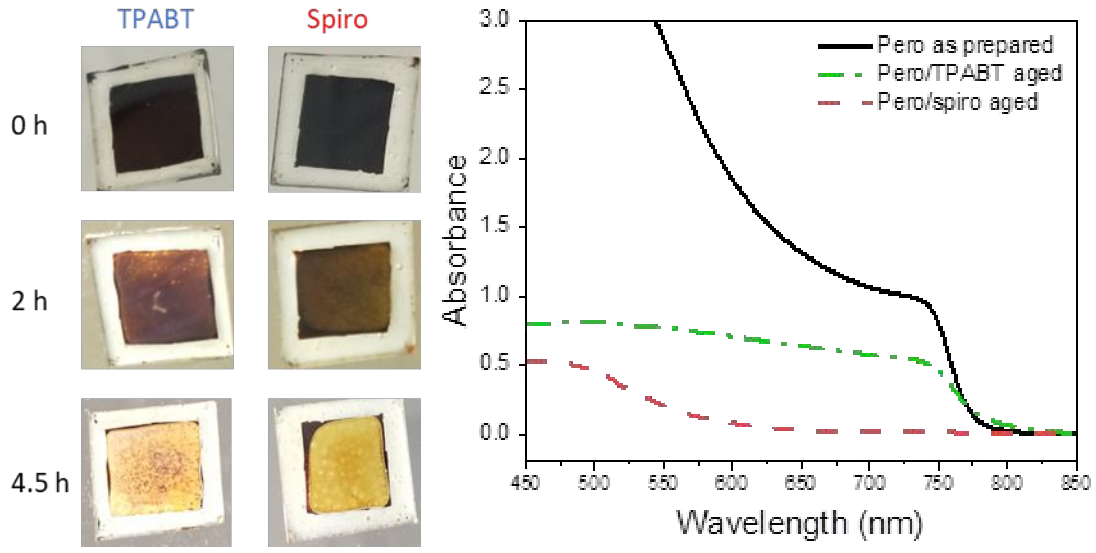

Figure 25. Left: visual appearance of the HTM-coated perovskite films before and after 2 hours and 4.5 hours exposure to moisture. Right: UV-visible absorption spectra before and after 4.5 hours exposure to moisture.

Exposing HTM coated perovskite films to a high humidity environment provided a rigorous stress toward moisture ingress. The steep onset at about 770 nm indicates the presence of perovskite films in the fresh sample (t = 0 hour). After 4.5 hours of aging, the TPABT coated perovskite films appeared to be darker visually corresponding to presence of perovskite meanwhile, the absorbance of Spiro-OMeTAD coated samples no longer possessed perovskite signatory absorbance profile.

References:

(1) Tortorella, S.; Talamo, M. M.; Cardone, A.; Pastore, M.; De Angelis, F. Benchmarking DFT and Semi-Empirical Methods for a Reliable and Cost-Efficient Computational Screening of Benzofulvene Derivatives as Donor Materials for Small-Molecule Organic Solar Cells. *J. Phys. Condens. Matter* **2016**, *28* (7), 074005. https://doi.org/10.1088/0953-8984/28/7/074005.

(2) Chi, W.-J.; Li, Q.-S.; Li, Z.-S. Exploring the Electrochemical Properties of Hole Transport Materials with Spiro-Cores for Efficient Perovskite Solar Cells from First-Principles.

Nanoscale **2016**, *8* (11), 6146–6154. https://doi.org/10.1039/C6NR00235H.

(3) Petrus, M. L.; Schutt, K.; Sirtl, M. T.; Hutter, E. M.; Closs, A. C.; Ball, J. M.; Bijleveld, J. C.; Petrozza, A.; Bein, T.; Dingemans, T. J.; Savenije, T. J.; Snaith, H.; Docampo, P. New Generation Hole Transporting Materials for Perovskite Solar Cells: Amide-Based Small-Molecules with Nonconjugated Backbones. *Adv. Energy Mater.* **2018**, *8* (32), 1801605. https://doi.org/10.1002/aenm.201801605.

(4) Petrus, M. L.; Sirtl, M. T.; Closs, A. C.; Bein, T.; Docampo, P. Hydrazone-Based Hole Transporting Material Prepared via Condensation Chemistry as Alternative for Cross-Coupling Chemistry for Perovskite Solar Cells. *Mol. Syst. Des. Eng.* **2018**, *3* (5), 734–740. https://doi.org/10.1039/C8ME00023A.

(5) Petrus, M. L.; Music, A.; Closs, A. C.; Bijleveld, J. C.; Sirtl, M. T.; Hu, Y.; Dingemans, T. J.; Bein, T.; Docampo, P. Design Rules for the Preparation of Low-Cost Hole Transporting Materials for Perovskite Solar Cells with Moisture Barrier Properties. *J. Mater. Chem. A* **2017**, *5* (48), 25200–25210. https://doi.org/10.1039/c7ta06452g.

(6) Huang, C.; Fu, W.; Li, C.-Z.; Zhang, Z.; Qiu, W.; Shi, M.; Heremans, P.; Jen, A. K.-Y.; Chen, H. Dopant-Free Hole-Transporting Material with a C 3 h Symmetrical Truxene Core for Highly Efficient Perovskite Solar Cells. *J. Am. Chem. Soc.* **2016**, *138* (8), 2528–2531. https://doi.org/10.1021/jacs.6b00039.

(7) Zhang, Y.; Zhang, H.; Liu, A.; Chen, C.; Song, W.; Zhao, J. Rate-Limiting O–O Bond Formation Pathways for Water Oxidation on Hematite Photoanode. *J. Am. Chem. Soc.* **2018**, *140* (9), 3264–3269. https://doi.org/10.1021/jacs.7b10979.

(8) Bi, D.; Xu, B.; Gao, P.; Sun, L.; Grätzel, M.; Hagfeldt, A. Facile Synthesized Organic Hole Transporting Material for Perovskite Solar Cell with Efficiency of 19.8%. *Nano Energy* **2016**, *23*, 138–144. https://doi.org/10.1016/j.nanoen.2016.03.020.

(9) Salunke, J.; Guo, X.; Lin, Z.; Vale, J. R.; Candeias, N. R.; Nyman, M.; Dahlström, S.; Österbacka, R.; Priimagi, A.; Chang, J.; Vivo, P. Phenothiazine-Based Hole-Transporting Materials toward Eco-Friendly Perovskite Solar Cells. *ACS Appl. Energy Mater.* **2019**, *2* (5), 3021–3027. https://doi.org/10.1021/acsaem.9b00408.

(10) Sathiyan, G.; Ranjan, R.; Ranjan, S.; Garg, A.; Gupta, R. K.; Singh, A. Dicyanovinylene and Thiazolo[5,4- d]Thiazole Core Containing D–A–D Type Hole-Transporting Materials for Spiro-OMeTAD-Free Perovskite Solar Cell Applications with Superior Atmospheric Stability. *ACS Appl. Energy Mater.* **2019**, *2* (10), 7609–7618. https://doi.org/10.1021/acsaem.9b01598.

(11) Pashaei, B.; Shahroosvand, H.; Ameri, M.; Mohajerani, E.; Nazeeruddin, M. K. A Sequential

Condensation Route as a Versatile Platform for Low Cost and Efficient Hole Transport Materials in Perovskite Solar Cells. *J. Mater. Chem. A* **2019**, *7* (38), 21867–21873. https://doi.org/10.1039/C9TA05121J.

(12) Magomedov, A.; Kasparavičius, E.; Rakstys, K.; Paek, S.; Gasilova, N.; Genevičius, K.; Juška, G.; Malinauskas, T.; Nazeeruddin, M. K.; Getautis, V. Pyridination of Hole Transporting Material in Perovskite Solar Cells Questions the Long-Term Stability. *J. Mater. Chem. C* **2018**, *6* (33), 8874–8878. https://doi.org/10.1039/C8TC02242A.

(13) Zhao, X.; Zhang, F.; Yi, C.; Bi, D.; Bi, X.; Wei, P.; Luo, J.; Liu, X.; Wang, S.; Li, X.; Zakeeruddin, S. M.; Grätzel, M. A Novel One-Step Synthesized and Dopant-Free Hole Transport Material for Efficient and Stable Perovskite Solar Cells. *J. Mater. Chem. A* **2016**, *4* (42), 16330–16334. https://doi.org/10.1039/C6TA05254A.

(14) Xu, B.; Zhang, J.; Hua, Y.; Liu, P.; Wang, L.; Ruan, C.; Li, Y.; Boschloo, G.; Johansson, E. M. J.; Kloo, L.; Hagfeldt, A.; Jen, A. K.-Y.; Sun, L. Tailor-Making Low-Cost Spiro[Fluorene-9,9′-Xanthene]-Based 3D Oligomers for Perovskite Solar Cells. *Chem* **2017**, *2* (5), 676–687. https://doi.org/10.1016/j.chempr.2017.03.011.

(15) Lee, D.-H.; Liu, Y.-P.; Lee, K.-H.; Chae, H.; Cho, S. M. Effect of Hole Transporting Materials in Phosphorescent White Polymer Light-Emitting Diodes. *Org. Electron.* **2010**, *11* (3), 427–433. https://doi.org/10.1016/j.orgel.2009.11.022.

(16) Röhr, J. A.; Moia, D.; Haque, S. A.; Kirchartz, T.; Nelson, J. Exploring the Validity and Limitations of the Mott–Gurney Law for Charge-Carrier Mobility Determination of Semiconducting Thin-Films. *J. Phys. Condens. Matter* **2018**, *30* (10), 105901. https://doi.org/10.1088/1361-648X/aaabad.

(17) Snaith, H. J.; Grätzel, M. Enhanced Charge Mobility in a Molecular Hole Transporter via Addition of Redox Inactive Ionic Dopant: Implication to Dye-Sensitized Solar Cells. *Appl. Phys. Lett.* **2006**, *89* (26), 262114. https://doi.org/10.1063/1.2424552.
